# Comparison of the efficacy and safety in the treatment strategies between chemotherapy combined with antiangiogenic and with immune checkpoint inhibitors in advanced non-small cell lung cancer patients with negative PD-L1 expression: A network meta-analysis

**DOI:** 10.3389/fonc.2022.1001503

**Published:** 2022-11-29

**Authors:** Jiaqi Li, Yingjie Chen, Fan Hu, Huiping Qiang, Qing Chang, Jialin Qian, Yinchen Shen, Yong Cai, Tianqing Chu

**Affiliations:** ^1^ Department of Respiratory Medicine, Shanghai Chest Hospital, Shanghai Jiaotong University, Shanghai, China; ^2^ School of Public Health, Shanghai Jiaotong University, Shanghai, China

**Keywords:** non-small cell lung cancer (NSCLC), immune checkpoint inhibitors (ICIs), programmed cell death ligand-1 (PD-L1), negative PD-L1 expression, antiangiogenic therapy, combination, survival analysis, network meta-analysis (NMA)

## Abstract

**Background:**

In the first-line treatment of advanced non-small cell lung cancer (NSCLC), for those patients with negative PD-L1 expression, which treatment strategy has the better efficacy and safety between chemotherapy combined with antiangiogenic and with immune checkpoint inhibitors (ICIs) is still unclear due to the absence of head-to-head clinical trials. This study aims to answer the question by performing a systematic review and network meta-analysis (NMA).

**Methods:**

Electronic databases (PubMed, Embase, Cochrane Library, Web of Science, and ClinicalTrials.gov) were systematically searched accordingly to extract eligible studies from inception to October 2022, as well as the abstracts from the most recent main oncology congresses (American Association for Cancer Research (AACR), American Society of Clinical Oncology (ASCO), World Conference on Lung Cancer (WCLC), and European Society for Medical Oncology (ESMO)). Overall survival (OS), progression-free survival (PFS), and adverse events (AEs) of grades 3 to 5 were independently extracted and collected by two reviewers based on the Preferred Reporting Items for Systematic Reviews and Meta-Analyses (PRISMA) guideline. We used Cochrane’s risk of bias tool for randomized controlled trials through RevMan 5.3 to ascertain the quality of the included studies. NMA with a Bayesian random-effects model was performed by R (version 4.0.4).

**Results:**

According to the ranking list from OS-NMA, pembrolizumab combined with chemotherapy has the most effective ranking first (surface under the cumulative ranking (SUCRA) = 0.809844) (pooled HR = 0.65 [0.51–0.83]). On PFS, the triple combination of nivolumab/bevacizumab/chemotherapy ranks first (NMA estimate: HR = 0.35 [0.28–0.43]). On safety, in combination with chemotherapy, sintilimab has minimal toxicity, followed by pembrolizumab+chemo.

**Conclusions:**

In advanced NSCLC patients with negative PD-L1 expression, pembrolizumab+chemo ranks first in the efficacy of OS and does not apparently increase the incidence of any grade ≥ 3 AE as compared with chemo alone. On PFS, pembrolizumab also has advantages, but for patients with squamous cell carcinoma, camrelizumab+chemo seems to be a better choice.

**Systematic review registration:**

https://www.crd.york.ac.uk/prospero/, identifier CRD42021231441.

## 1 Background

Lung cancer is the main cause of cancer death, and its incidence rate is more than 1/10 of the world’s malignant tumors (1). Advanced non-small cell lung cancer accounts for more than two-thirds of the patients. Due to the lack of effective treatment, the survival rate had been low (2). With the emergence of tumor antiangiogenesis drugs, the survival time of patients with advanced lung cancer finally exceeded 1 year. Originating from ECOG4599, the non-small cell lung cancer (NSCLC) patients treated with the combination of bevacizumab with carboplatin and paclitaxel survived longer than those who received chemotherapy alone (overall survival (OS)) (12.3 vs. 10.3 months, HR = 0.79, 95% CI [0.67–0.92], *p* = 0.003); median progression-free survival (PFS) (6.2 vs. 4.5 months, HR = 0.66, 95% CI [0.57–0.77], *p* < 0.001) (3). Because of this study, bevacizumab combined with paclitaxel and carboplatin was approved by the Food and Drug Administration (FDA) in 2006 as the first-line standard treatment for advanced non-small cell lung cancer. The BEYOND study confirmed that bevacizumab combined with chemotherapy can prolong OS to 24.8 months and the overall response rate (ORR) to 53.4% in the Chinese population (4).

In the past decade, the field of therapeutic strategies for NSCLC has acquired a completely new outlook since the emergence of targeted agents directed at specific driver mutations and immune checkpoint inhibitors (ICIs) and showed great potential. Longer survival and higher disease response rate have been seen in metastatic NSCLC patients who are treated with checkpoint inhibitors targeting programmed cell death protein 1 (PD-1) and programmed death ligand-1 (PD-L1) (5). Keynote024 and Impower110 studies showed that in patients with PD-L1 expression of more than 50%, ICI monotherapy can prolong OS of patients with advanced NSCLC in the first-line treatment. The combinations of ICIs and chemotherapy allow more patients to achieve better ORR and prolonged OS regardless of PD-L1 expression level and have become a standard first-line treatment for advanced NSCLC (6, 7). Non-squamous NSCLC patients with any level of PD-L1 expression were enrolled in the keynote-189 study, and the results showed that the efficacy of pembrolizumab+chemotherapy was superior to that of chemotherapy alone. However, when we consider PD-L1 expression level, the ORR was 62.1%, 50.0%, and 33.1% in patients with greater than 50% PD-L1 expression, 1%–49% PD-L1 expression, and <1% PD-L1 expression, respectively; OS was 27.7, 21.8, and 17.2 months, respectively; PFS was 11.1, 9.4, and 6.2 months, respectively (8). As we have seen in other studies of ICIs combined with chemotherapy, the improvement in objective response rate and prolongation of survival with combination therapies were less pronounced in patients negative for PD-L1 expression than in those positive for PD-L1. As previously described in the literature (9), the biological mechanisms of the immune response are complex, and the efficacy of PD-L1 immune checkpoint inhibitors cannot be simply measured by the direct pharmacodynamic effect of binding PD-L1. Biomarkers remain controversial, especially in different histological types. Therefore, it is an interesting topic to study the response of patients with negative PD-L1 expression to different ICIs. Furthermore, chemotherapy combined with antiangiogenic agents or ICIs both are the first-line standards for the treatment of advanced NSCLC. The question is which of the two strategies is more advantageous for patients with negative PD-L1 expression? Due to the lack of head-to-head clinical evidence, the answer to this question is unclear (5, 10). Therefore, our study aims to explore a better treatment strategy between antiangiogenic combined with chemotherapy and immune checkpoint inhibitors combined chemotherapy in advanced NSCLC patients with negative PD-L1 expression by performing a systematic review and network meta-analysis (NMA).

We present the following article in accordance with the Preferred Reporting Items for Systematic Reviews and Meta-Analyses (PRISMA) reporting guidelines.

## 2 Methods

The specified analysis methods and inclusion criteria are described below, and the study adheres to the PRISMA extension statement for systematic reviews incorporating NMAs (11). This study was registered with PROSPERO (ID: CRD 42021231441) (https://www.crd.york.ac.uk/prospero/).

### 2.1 Data acquisition

#### 2.1.1 Eligibility criteria

Studies were considered if they met the inclusion criteria, as follows: I) prospective randomized controlled trials (RCTs; II) patients with histologically proven diagnosis of advanced NSCLC; III) randomized phase-III studies with at least one treatment with ICI/angiogenesis inhibitors combined with platinum-based chemotherapy among patients with no prior systemic anticancer therapies in the first-line settings; IV) studies showing efficacy outcomes, including OS and PFS, and the reported results included the PD-L1-negative expression level (PD-L1 < 1%); V) studies displayed safety profiles, including the incidence of grade ≥ 3 adverse events (AEs). Furthermore, the following studies were excluded to minimize the risk of bias: I) unrandomized studies, phase I and phase II studies, single-arm studies, retrospective studies, reviews, meta-analyses, case reports, letters, and comments; II) studies in which the comparison regimens were in combination with targeted therapy or radiotherapy; III) lacking necessary data or overlapped studies.

#### 2.1.2 Information sources, search strategies, and study selection

**Figure 1:**
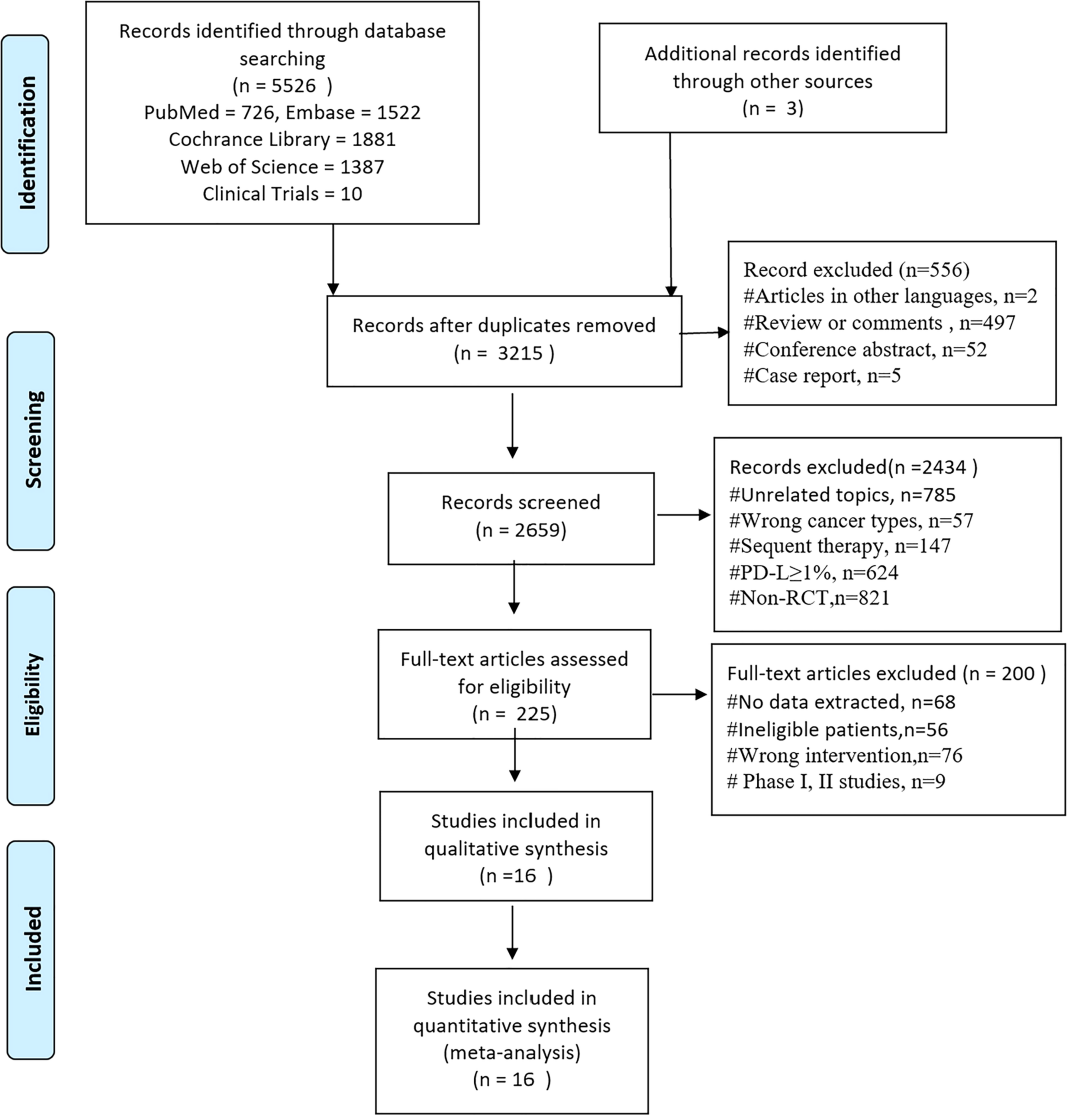
PRISMA diagram of search results and selections. PRISMA, Preferred Reporting Items for Systematic Reviews and Meta-Analyses.

Studies were identified by searching Pubmed, Embase, Cochrane Library, and Web of Science (retrieval date 4 October 2022), with a language restriction of English. The following groups of keywords and medical terms were used: (“Carcinoma*, Non?Small?Cell Lung” or “Lung Carcinoma*, Non-Small-Cell” or “Non?Small?Cell Lung Cancer” or “NSCLC”) and (“PD-1” or “PD-L1” or “Pembrolizumab” or “Nivolumab” or “Atezolizumab” or “Camrelizumab” or “Tislelizumab” or “Sintilimab” or “Tremelimumab” or “Ipilimumab” or “Ticilimumab” or “Durvalumab” or “Inhibitor*, Angiogen*” or “Antagonist* Angiogen*” or “Angiostatic Agent*” or “Anti?Angiogen* Agent*” or “Anti?Angiogen* Drug*” or “Inhibitor*, Neovascularization” or “VEGFR” or “multitargeted antiangiogenesis tyrosine kinase inhibitors” or “bevacizumab” or “ramucirumab” or “nintedanib” or “anlotinib” or “apatinib” or “Vandetanib” or “Sunitinib” or “Pazopanib” or “Lenvatinib” or “Cediranib” or “Motesanib” or “Axitinib”) and (“chemotherap*” or “chemotherapy” or “Platinum” or “Carboplatin” or “cisplatin”) and (randomized controlled trial or randomized or RCT). The abstracts in the most recent main oncology congresses [American Association for Cancer Research (AACR), American Society of Clinical Oncology (ASCO), World Conference on Lung Cancer (WCLC), and European Society for Medical Oncology (ESMO); retrieval date 4 October 2022)], for related phase III clinical trials identified in the “clinicaltrials.gov” site (**Figure 1**), were also used. The specific search strategy is in **Supplementary Table S1**.

#### 2.1.3 Assessment of risk of bias

To ascertain the quality of the studies included in the current NMA, we used Cochrane’s risk of bias tool for randomized controlled trials, in which six aspects were evaluated: random sequence generation, allocation concealment, blinding of participants and personnel, blinding of outcome assessment, incomplete outcome data, selective reporting, and other sources of bias (12).

### 2.2 Statistical analysis

The data were analyzed through direct pairwise comparisons, and the results were presented with pooled-estimated hazard ratios (HRs pooled) together with 95% CIs for the first step. Mixed treatment comparisons incorporating direct and indirect results were subsequently generated within Bayesian frameworks using Markov chain Monte Carlo methods. The primary endpoint of interest in the NMA is OS in the overall study; PFS and safety were examined simultaneously.

The analyses above were performed by R (version 4.0.4) as well as R packages (https://www.r-project.org/). In the R package “gemtc”, each chain was fitted with 20,000 iterations. Afterward, different treatments were ranked by efficacy and safety outcomes by calculating the HRs or odds ratios (ORs) as well as the proportion of iterations of the Markov chain. The surface under the cumulative ranking (SUCRA) of treatments was estimated to determine the likelihood of therapies in a best-to-worst order. The value of SUCRA is between 0 and 1 (0 ≤ SUCRA ≤ 1). When SUCRA is 1, it indicates that the intervention is absolutely effective, and when it is 0, it indicates that the intervention is absolutely ineffective.

### 2.3 Heterogeneity and sensitivity analyses

Random-effects models were used due to inherent clinical heterogeneity in the study data. Heterogeneity between studies was measured by the I^2^ test and *p*-value. I^2^ with values of 25%, 50%, and 75%, respectively indicating low, moderate, and high heterogeneity. Two-tailed *p*-values <0.05 were considered to indicate statistical significance (13).

In the comparison of efficacy, we conducted two sensitivity analyses due to the significant heterogeneity (I^2^ ≥ 75%) in the chemotherapy combined with bevacizumab or combined with atezolizumab groups. We removed the BEYOND study (4) from the former analysis and removed the IMpower132 study (14) from the second sensitivity analysis. Pooled efficacy was compared again after the omissions (15). On the safety comparison, we conducted one sensitivity analysis due to the significant heterogeneity (I^2^ ≥75%) in the chemotherapy-combined camrelizumab.

## 3 Results

### 3.1 Studies included in the network meta-analysis

A total of 5,526 records were obtained through a literature search in our study. After a full-text review of 225 articles, we identified 16 trials for qualitative and quantitative syntheses (**Figure 1**) (3, 4, 8, 14, 16–31), and four of them were conference abstracts or presentations (16, 18, 19, 27). For data from the same study, only the latest and most comprehensive update can be included in the NMA.

### 3.2 Study characteristics

The characteristics of included studies are summarized in **Table 1**. In 16 RCTs, controls received only chemotherapy, with the exception of two studies in which bevacizumab was added to both the trial and control groups [IMPower150, TASUKI-52 (19) (31)]. ICI in combination with chemotherapy was tested in 12 studies involving eight treatment strategies (pembrolizumab, two; atezolizumab, four, one with/without bevacizumab; nivolumab, two, one with/without bevacizumab; sintilimab, one; camrelizumab, two; and tislelizumab, one), and angiogenesis inhibitors combined with chemotherapy were tested in four studies involving three treatments (bevacizumab, two; motesanib, one; and cediranib, one; In bevacizumab combination chemotherapy, the chemotherapy regimens have prescribed a limit to carboplatin/cisplatin+paclitaxel).

**Table 1:** (A) Characteristics of studies (ICI+chemo) included in network meta-analysis.

Study	ClinicalTrials.gov Identifier	Histology	Arm	No.pts	PD-L1<1%				AE(≥grade3)of any cause(%pts)
					No.pts	HR OS (95%CI)	HR PFS (95%CI)	Analysis Timing
IMpower131	NCT02367794	Squamous	Atezolizumab+Chemo	343	160	0.87 (0.67,1.13)	0.82(0.65,1.04)	PFS:Final	83%
			Chemo	340	171			OS:Final	70.40%
IMpower130	NCT02367781	Non-Squamous	Atezolizumab+Chemo	451	235	0.81 (0.61,1.08)	0.72 (0.56,0.91)	PFS:Final	85.80%
			Chemo	228	121			OS:Final	76.30%
IMpower132	NCT02657434	Non-Squamous	Atezolizumab+Chemo	292	88	0.67 (0.46,0.96)	0.45 (0.31,0.64)	PFS:Final	71.50%
			Chemo	286	75			OS:Final	60.60%
KEYNOTE-189	NCT03950674	Non-Squamous	Pembrolizumab+Chemo	410	127	0.52 (0.36,0.74)	0.64 (0.47,0.89)	PFS:Final	71.90%
			Chemo	206	63			OS:Final	66.80%
KEYNOTE-407	NCT03875092	Squamous	Pembrolizumab+Chemo	278	95	0.79 (0.56,1.11)	0.67(0.49,0.91)	PFS:Final	74.10%
			Chemo	281	99			OS:Final	69.60%
CheckMate 227	NCT02477826	All	Nivolumab+Chemo	377	177	0.82 (0.65,1.02)	0.72(0.57,0.91)	PFS:Final	58.10%
			Chemo	378	186			OS:Final	37.10%
IMpower150	NCT02366143	Non-Squamous	ArmA: Atezo+Chemo	349	164	A:C 0.96 (0.76,1.22)	NA	PFS:Final	60%
			ArmB: Atezo+Beva+Chemo	359	167	B:C 0.9 (0.71,1.14)	0.77 (0.61,0.99)	OS:Final	68%
			ArmC: Beva+Chemo	337	172				63%
ORIENT-11	NCT03607539	Non-Squamous	Sintilimab + Chemo	266	85	NA	0.664 (0.406,1.086)	PFS:Final	61.70%
			Chemo	131	44				58.80%
CameL-sq	NCT03668496	Squamous	Camrelizumab+Chemo	193	91	0.62 (0.41,0.94)	0.49 (0.35,0.68)	PFS:Final	74%
			Chemo	196	97				72%
CameL	NCT03134872	Non-Squamous	Camrelizumab+Chemo	205	49	NA	0.76 (0.45,1.26)	PFS:Final	69%
			Chemo	207	69				47%
RATIONALE307	NCT05024266	Squamous	Tislelizumab+Chemo	120	48	NA	0.636 (0.368,1.1)	PFS:Interim	88.30%
			Chemo	121	49	NA			83.80%
TASUKI-52	NCT03117049	Non-Squamous	Nivolumab+Beva+Chemo	275	120	NA	0.55 (0.38,0.78)	PFS:Interim	75.50%
			Beva+Chemo	275	120	NA			73.50%

OS, overall survival; PFS, progress free survial.

**Table 1:** (B) Characteristics of studies (Antiangiogenic drugs+Chemo) included in network meta-analysis.

Study	ClinicalTrials.gov Identifier	Histology	Arm	No.pts	OS HR (95%CI)	PFS HR (95%CI)	Analysis Timing	AE(≥grade3)of any cause(%pts)
EYOND	NCT01364012	Non-Squamous	Bevacizumab + Chemo	138	0.68 (0.5,0.93)	0.4 (0.29,0.54)	PFS:Final	67%
			Chemo	138			OS:Final	62.00%
ECOG-4599	NCT00021060	Non-Squamous	Bevacizumab + Chemo	434	0.79 (0.67,0.92)	0.66 (0.57,0.77)	PFS:Final	NA
			Chemo	444			OS:Final	NA
MONET1	NCT00460317	Non-Squamous	Motesanib+Chemo	541	0.9 (0.78,1.04)	0.79 (0.68,0.9)	PFS:Final	73.00%
			Chemo	549			OS:Final	59%
BR29	NCT00795340	All	Cediranib+Chemo	153	0.94 (0.69,1.3)	0.91 (0.71,1.18)	PFS:Final	NA
			Chemo	153			OS:Final	NA

OS, overall survival; PFS, progress free survial.

### 3.3 Assessment of risk of bias

All studies included in this NMA were considered of low risk of bias based on the quality evaluation results accessing Cochrane’s tool for randomized trials. Five of the studies were deemed to be at high risk of performance for the reason of being open-label. Random sequence generation and complete outcomes were reported in every article, while some of them did not mention allocation concealment and the blinding in outcome access (**Supplementary Figure S1**).

### 3.4 Efficacy evaluation: overall survival

**Figure 2:**
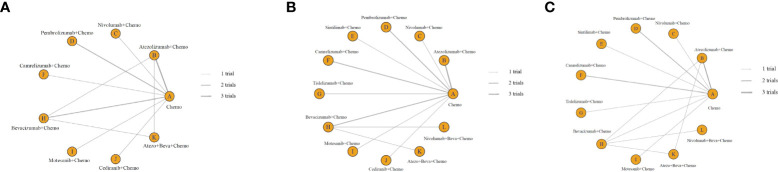
**(A)** Network of OS. **(B)** Network of PFS. **(C)** Network of safety. OS, overall survival; PFS, progression-free survival.

The OS-NMA for the overall study cohort covers 12 of the 16 studies with available OS information involving nine treatments that are both squamous and non-squamous (**Figure 2A**). Among them, three studies about atezolizumab+chemotherapy (chemo), pembrolizumab+chemotherapy, and bevacizumab+chemotherapy each consist of two studies. The remaining treatments each include one study.

**Figure 3:**
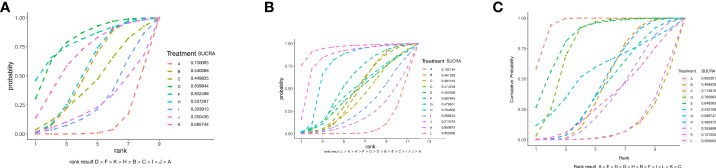
**(A)** Cumulative ranking plot for OS. **(B)** Cumulative ranking plot for PFS. **(C)** Cumulative ranking plot for safety. OS, overall survival; PFS, progression-free survival.

On the basis of the ranking list from NMA, the top four of all treatments in terms of efficacy for OS were pembrolizumab+chemo, atezo+beva+chemo, bevacizumab+chemo, and atezolizumab+chemo. Pembrolizumab combined with chemotherapy seems to be the most effective and ranks first in PD-L1-negative patients (SUCRA = 0.809844) (**Figure 3A**).

The pooled HR of every treatment can be seen in the forest plot (**Supplementary Figures S4A**) and **Table 2**. The combination of chemotherapy with pembrolizumab (pooled HR = 0.65, 95% CI [0.51–0.83]) or atezolizumab (pooled HR = 0.78, 95% CI [0.68–0.90]) or atezolizumab+bevacizumab (HR = 0.72, 95% CI [0.57–0.91]) or bevacizumab (pooled HR = 0.78 [0.69–0.88]) showed a significant benefit in OS over chemotherapy alone.

**Table 2:** Pooled hazard ratios (95% credible intervals) for progression-free survival (lower triangle) and overall survival (upper triangle).

	Overall survival											
**Progression-Free-Survival**	A	**0.78 (0.68, 0.90)**	0.82 (0.65, 1.03)	**0.65 (0.51, 0.83)**	-	**0.62 (0.41, 0.94)**	-	**0.78 (0.69, 0.88)**	0.9 (0.78, 1.04)	0.94 (0.69, 1.29)	**0.72 (0.57, 0.91)**	-
	**0.70 (0.60, 0.82)**	B	1.05 (0.80, 1.37)	0.83 (0.62, 1.1)	-	0.79 (0.51, 1.23)	-	0.99 (0.85, 1.17)	1.15 (0.94, 1.41)	1.20 (0.85, 1.7)	0.92 (0.73, 1.14)	-
	**0.72 (0.61, 0.85)**	1.03 (0.82, 1.29)	C	0.79 (0.57, 1.10)	-	0.76 (0.47, 1.21)	-	0.95 (0.73, 1.23)	1.10 (0.84, 1.43)	1.15 (0.78, 1.69)	0.87 (0.63, 1.21)	-
	**0.66 (0.53, 0.82)**	0.94 (0.71, 1.23)	0.91 (0.69, 1.20)	D	-	0.96 (0.59, 1.55)	-	1.2 (0.91, 1.59)	1.39 (1.04, 1.85)	1.45 (0.97, 2.17)	1.11 (0.79, 1.55)	-
	0.66 (0.41, 1.09)	0.95 (0.57, 1.59)	0.92 (0.55, 1.55)	1.01 (0.59, 1.74)	E	-	-	-	-	-	-	-
	**0.56 (0.42, 0.74)**	0.80 (0.58, 1.09)	0.77 (0.56, 1.07)	0.85 (0.59, 1.21)	0.84 (0.48, 1.48)	F	-	1.25 (0.81, 1.94)	1.45 (0.94, 2.25)	1.52 (0.90, 2.56)	1.15 (0.72, 1.86)	-
	0.64 (0.37, 1.10)	0.91 (0.52, 1.61)	0.88 (0.5, 1.57)	0.97 (0.54, 1.75)	0.96 (0.46, 2.00)	1.14 (0.62, 2.11)	G	-	-	-	-	-
	**0.63 (0.57, 0.69)**	0.90 (0.75, 1.08)	0.87 (0.72, 1.06)	0.96 (0.75, 1.22)	0.95 (0.57, 1.57)	1.13 (0.84, 1.52)	0.99 (0.57, 1.72)	H	1.16 (0.95, 1.40)	1.21 (0.86, 1.70)	0.92 (0.74, 1.15)	-
	0.81 (0.64, 1.03)	1.16 (0.87, 1.53)	1.12 (0.84, 1.51)	1.24 (0.89, 1.71)	1.22 (0.71, 2.11)	**1.45 (1.01, 2.10)**	1.27 (0.70, 2.31)	1.29 (1.00, 1.66)	I	1.04 (0.74, 1.48)	0.80 (0.60, 1.05)	-
	0.91 (0.71, 1.17)	1.30 (0.97, 1.75)	1.26 (0.93, 1.72)	1.39 (0.99, 1.95)	1.37 (0.79, 2.39)	**1.63 (1.12, 2.38)**	1.43 (0.78, 2.62)	**1.45 (1.10, 1.90)**	1.12 (0.79, 1.59)	J	0.76 (0.51, 1.13)	-
	**0.48 (0.37, 0.63)**	**0.69 (0.51, 0.94)**	**0.67 (0.49, 0.92)**	0.74 (0.53, 1.04)	0.73 (0.42, 1.27)	0.87 (0.59, 1.27)	0.76 (0.42, 1.40)	**0.77 (0.60, 0.98)**	**0.60 (0.42, 0.85)**	**0.53 (0.37, 0.77)**	K	-
	**0.35 (0.28, 0.43)**	**0.50 (0.38, 0.65)**	**0.48 (0.36, 0.64)**	**0.53 (0.39, 0.72)**	**0.52 (0.30, 0.90)**	**0.62 (0.44, 0.89)**	**0.54 (0.30, 0.98)**	**0.55 (0.45, 0.67)**	**0.43 (0.31, 0.59)**	**0.38 (0.27, 0.53)**	**0.71 (0.52, 0.98)**	L

Bold values represent statistically significant HR values.

Dark blue indicates the treatment strategy involved in the study, light blue indicates pooled HR and 95% confidence interval for OS, and colorless indicates pooled HR and 95% confidence interval for PFS.

### 3.5 Efficacy evaluation: progression-free survival

The 16 studies included in the network analysis all provided PFS information (**Figure 2B**; **Table 1**). Three of the studies evaluated atezolizumab+chemo. The combination of chemotherapy with pembrolizumab or camrelizumab contains two studies. The other treatments each consist of one study.

Based on the NMA estimates, the triple combination of nivolumab/bevacizumab/chemotherapy is estimated to be better than all other treatments evaluated in PFS, ranking first (SUCRA = 0.952696), followed by atezo+beva+chemo, bevacizumab+chemo, camrelizumab+chemo, tislelizumab+chemo, pembrolizumab+chemo, atezolizumab+chemo, etc. (**Figure 3B**). Among them, chemotherapy combined with camrelizumab or pembrolizumab (pooled HR = 0.56 [0.42–0.74] or pooled HR = 0.66 [0.53–0.82], respectively) or atezolizumab (with/without bevacizumab) (NMA estimate: HR = 0.48 [0.37–0.63]; pooled HR = 0.70 [0.60–0.82]), or nivolumab (with/without bevacizumab) (NMA estimate: HR = 0.35 [0.28–0.43]; pooled HR = 0.72 [0.61–0.85]) as well as the bevacizumab+chemo (pooled HR = 0.63 [0.57–0.69]) showed a significant PFS benefit over chemo alone, according to the forest plot (**Supplementary Figure S4B**) and **Table 2**.

### 3.6 Safety results

Fourteen studies included in the network analysis provided safety information (**Figure 2C**). The available incidence of AE (≥ grade 3) of any cause in each study is presented (**Table 1**).

The ranking results estimated by NMA show that in terms of safety, chemo alone ranks top one, followed by sintilimab+chemo, pembrolizumab+chemo, tislelizumab+chemo, bevacizumab+chemo, and atezolizumab+chemo (**Figure 3C**). Among all of these, sintilimab+chemo ((NMA estimate: pooled OR = 1.1 [0.73–1.70], pembrolizumab+chemo (pooled OR = 1.3 [0.97–1.60]), and tislelizumab+chemo (NMA estimate: pooled OR = 1.5 [0.73–3.20]) do not apparently increase the incidence of any grade ≥ 3 AE compared with chemo alone (**Supplementary Figure S4C**).

### 3.7 Combination of efficacy and safety

**Figure 4:**
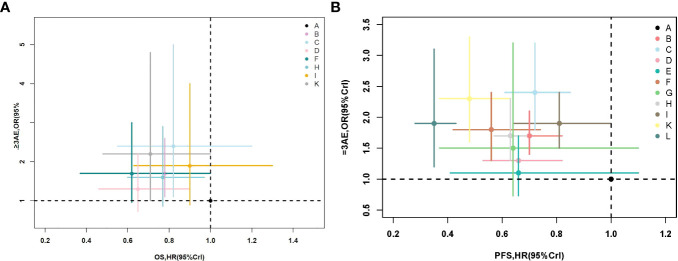
**(A)** Efficacy (on OS) and safety (≥3 AE). **(B)** Efficacy (on PFS) and safety (≥3 AE). OS, overall survival; AE, adverse event; PFS, progression-free survival.

From **Figures 4A, B**, we can see that considering the efficacy of OS and safety, chemotherapy combined with pembrolizumab is the best treatment strategy. It has the best curative effect for OS and does not increase toxicity as compared to chemotherapy alone. For PFS, the three-drug combination regimen can obtain better efficacy but increases the incidence of adverse events (≥3 grade). Compared with chemotherapy alone, pembrolizumab+chemo can significantly prolong PFS without increasing the incidence of adverse events (≥3 grade).

### 3.8 Heterogeneity and inconsistency assessment

Forest plots of pairwise comparisons with heterogeneity estimates for efficacy and safety were generated in **Supplementary Figure S2**. Our assessment suggested low or moderate heterogeneity except for three treatments. Sensitivity analyses were conducted in case of high heterogeneity.

The fit of the consistency model was similar to or better than that of the inconsistency model. Inconsistency between direct and indirect estimates from the node splitting analysis did not show significant differences (**Supplementary Figure S3**).

### 3.9 Sensitivity analysis

Sensitivity analyses were conducted on PFS and safety. After the removal of the BEYOND study (4) in the first sensitivity analysis, bevacizumab+chemotherapy was ranked behind pembrolizumab+chemo. After omitting IMpower132 (13) in the second sensitivity analysis, the ranking of atezolizumab+chemo also changed. After removing the CameL study in the sensitivity analysis, the ranking of camrelizumab+chemotherapy changed from seventh to second, which is behind chemo. The combination of chemotherapy with camrelizumab does not apparently increase the incidence of any grade ≥ 3 AE compared with chemo alone (pooled OR = 1.1 [0.71–1.80]). We explain the results of the sensitivity analyses in the “Discussion” section of the article.

## 4 Discussion

As far as we know, this is the most comprehensive network meta-analysis to date. We explore the ranking of efficacy and safety in the treatment strategies, including antiangiogenic combined with chemotherapy and immune checkpoint inhibitors combined with chemotherapy in advanced NSCLC patients with negative PD-L1 expression. In the NMA, we included 16 studies involving a total of 12 first-line treatments, which cover seven ICIs and three antiangiogenic drugs. The study results provide a reference for physicians to choose treatment strategies in clinical practice.

Our results found that considering the efficacy of OS and safety, pembrolizumab combined with chemotherapy is the number one treatment strategy (including the triple combination of atezolizumab/bevacizumab/chemotherapy) in patients with negative PD-L1 expression. It ranks first on the efficacy of OS and does not increase the hazard of no less than three adverse events (≥3 AE) compared with chemo alone. The top four treatments in terms of efficacy for OS were pembrolizumab+chemo, atezo+beva+chemo, bevacizumab+chemo, and atezolizumab+chemo. The PD-L1 inhibitor atezolizumab+chemo seems less effective compared with bevacizumab+chemo, and both are significantly better than chemo alone. Why does anti-PD-L1 mAb/anti-PD-1 mAb therapy still show an obvious clinical response in the absence of PD-L1 expression on tumor cells? Why does pembrolizumab seem more effective than atezolizumab?

As previously reported, the biological mechanisms of the immune response are complex, and the efficacy of PD-L1 immune checkpoint inhibitors cannot be simply responded to by the direct pharmacodynamic effects of binding PD-L1; other immune mediators may also be involved in the immune response driven by ICIs and mediated only in part by PD-L1 overexpression, for example, tumor immunogenicity (9). Some preliminary studies have shown that tumors with high-load somatic mutations are more likely to respond to immunotherapy by presenting neoepitopes that may behave as neoantigens (32). To test this hypothesis, Snyder et al. performed whole-exome sequencing of tumor samples from melanoma patients treated with ipilimumab and pembrolizumab. As expected, high somatic mutation load correlated with response to treatment in most patients, and the quality, rather than the number, of mutations, was likely to have the strongest predictive value (33). Moreover, PD-L1 expresses not only on tumor cells but also on immune cells in the tumor microenvironment, such as T cells, natural killer (NK) cells, and macrophages (34). A study in acute myeloid leukemia (AML) revealed the mechanism of the efficacy of anti-PD-L1 mAb against PD-L1-negative tumors. The study found that tumor cells can induce PD-L1 expression on NK cells *via* the PI3K/AKT/NFκB pathway. Anti-PD-L1 mAb binds to the PD-L1 receptor on NK cells to activate NK cells. Activated NK cells exert powerful cytotoxic effects through multiple mechanisms such as perforin, granzyme B, tumor necrosis factor-related apoptosis-inducing ligand (TRAIL), or factor-related apoptosis ligand (FASL). In addition, interferon-gamma (IFNγ) produced by NK cells can directly affect target cells and activate macrophages and T cells to kill tumor cells or enhance the anti-tumor activity of other immune cells (35, 36). The cytotoxicity and ability to kill cancer cells of NK cells have been confirmed in many types of tumors, including lung cancer (37). These may provide a potential explanation as to why some patients with PD-L1-negative expression on tumor cells still respond to anti-PD-L1 mAb therapy. In addition, some factors will impact the accuracy of PD-L1 detection, and a false-negative occurrence may affect the results of our meta-analysis.

As for why pembrolizumab seems more effective than atezolizumab in our study, considering pembrolizumab is a human IgG4 mAb against PD-1 and atezolizumab is a humanized “anti-programmed death-ligand 1 (PD-L1) mAb”, we speculate that in patients with negative PD-L1 expression, PD-1 inhibitors are more effective than PD-L1 inhibitors. A previous meta-analysis by Professor Wang Jie indicated that patients obtained greater OS benefits from treatments containing anti-PD-1 compared with anti-PD-L1 in NSCLC and gastric carcinoma (38), which supports our results. However, the previous study did not perform subgroup analysis on the expression status of PD-L1 in tumor tissues, so our results supplement it. One possible reason for this difference is that PD-1 mAbs not only block the PD-1/PD-L1 axis but also block the PD-1/PD-L2 axis, inhibiting the tumor’s immune evasion mechanism more thoroughly, while PD-L1 mAbs only blocks PD-L1. Another possible reason is that PD-L1 mAb has a larger molecular weight and more robust immunogenicity. It produces more anti-drug antibodies (ADAs) in the body, which will reduce the efficacy of PD-L1. These might be reasons why the efficacy of atezolizumab+chemo is inferior to that of pembrolizumab/bevacizumab+chemo in advanced NSCLC patients with negative PD-L1 expression.

The ranking results on the efficacy of PFS are different from those of OS. Bevacizumab combined with chemotherapy (platinum and paclitaxel) ranks better than pembrolizumab combined with chemotherapy followed by camrelizumab+chemo, tislelizumab+chemo, pembrolizumab+chemo, atezolizumab+chemo, etc. This is because immunotherapy relatively responds slowly; in the short term, the advantages of ICIs+chemo vs. chemo are not as apparent as those of bevacizumab+chemo vs. chemo. However, the reaction of immunotherapy will last over time due to immunological memory (39). In addition, hyperprogressive disease (HPD), which is characterized by the acceleration of tumor growth, will occur during immunotherapy. This type of progression partially explains the crossover between survival curves observed in some clinical trials during the first months of treatment (39, 40). Some patients do not benefit from immunotherapy in the short term, which affects the PFS efficacy of ICIs.

In the first sensitivity analysis, when the BEYOND study (4) is removed, the ranking of bevacizumab+chemo changed behind pembrolizumab+chemo. Considering that all the patients enrolled in the BEYOND study were Chinese, while most of the patients enrolled in ECOG4599 were white, we speculate that race may be one of the reasons for the heterogeneity. Bevacizumab+chemo has a better curative effect, as shown in the BEYOND study, so Chinese patients seem to benefit more from bevacizumab on PFS compared with western patients.

In the second sensitivity analysis, after removing the IMpower132 study (13), the change may be related to the different maintenance treatments in the two clinical trials. In IMpower132, patients in the atezolizumab+chemotherapy group received maintenance treatment with atezolizumab+pemetrexed. In IMpower130, the patients received pemetrexed monotherapy as maintenance therapy. The results of the sensitivity analysis suggest that the group that received atezolizumab+pemetrexed as maintenance therapy may have a longer progression-free survival.

In the third sensitivity analysis, when the CameL study is removed, the ranking of camrelizumab+chemo changed from the seventh to the second. The combination of chemotherapy with camrelizumab does not apparently increase the incidence of any grade ≥ 3 AE compared with chemo alone (pooled OR = 1.1 [0.71–1.80]), after removing the CameL study. Considering that all the patients enrolled in the CameL study were non-squamous while all of the patients enrolled in CameL-sq were squamous, we speculate that histology type may be one of the reasons for the heterogeneity. Camrelizumab+chemo has better safety as shown in the CameL-sq study, so patients with squamous cell carcinoma maybe have a higher safety profile of using camrelizumab compared with patients with non-squamous cell carcinoma.

Additionally, in the CameL study (21), camrelizumab+chemo did not appear to confer PFS benefit when compared with chemotherapy alone in patients with PD-L1-negative expression (HR = 0.76 [0.45–1.26]), whereas it became the third preferred treatment in our NMA (HR = 0.56 [0.42–0.74]). Camrelizumab as the better therapeutic effect in squamous cell carcinoma could be the cause.

In CameL-sq study (27), camrelizumab+chemo performed better than chemo in PD-L1-negative patients (HR = 0.49 [0.35–0.68]). Therefore, in squamous non-small cell lung cancer, camrelizumab+chemo has a high probability of a superior PFS better than pembrolizumab+chemo (HR = 0.67 [0.49–0.91]) in KRYNOTE-407 (18), and its OS results (HR = 0.62 [0.41–0.94]) are also better than those of pembrolizumab+chemo (HR = 0.79 [0.56–1.11]) in KRYNOTE-407. Therefore, in squamous carcinoma, the efficacy and safety profile of camrelizumab were superior to those of non-squamous carcinoma, consistent with previous literature suggesting that PD-L1 expression levels may not be predictive of the efficacy of immunotherapy in patients with squamous carcinoma. The hypothesis that the impact of a rich cohort of coexisting mutations (as in the squamous subtype) may overcome the predictive power of PD-L1 is to be considered (9).

The advantages of this NMA are as follows. As the most comprehensive network meta-analysis by far, it evaluates and compares the efficiency and safety of chemotherapy combined with immune checkpoint inhibitors or with antiangiogenic therapy in advanced NSCLC patients with negative PD-L1 expression. In order to ensure that the entirety and quality of public results are available for the NMA, all phase III randomized clinical trial articles and conference summaries to date providing PD-L1-negative information were included. Moreover, to avoid bias in the interim report, the overall survival data were extracted at the final analysis of every trial. Inevitable limitations still existed in our study. First, we did not distinguish histological types, which could cause heterogeneity and unstable results on safety. Second, the PD-L1 detection methods and reagents vary from study to study, which may bring about deviations in baseline data. Finally, OS and the incidence of more than three AEs were not reported in each study. More head-to-head clinical studies are needed to verify the conclusion of this NMA based on the indirect comparison.

## 5 Conclusion

Based on the results of the NMA and approved indications of the combination treatment strategies, we found that in advanced NSCLC patients with negative PD-L1 expression, pembrolizumab+chemo ranks first in the efficacy of OS and does not apparently increase the incidence of any grade ≥ 3 AE as compared with chemo alone. On the efficacy of PFS, pembrolizumab was also able to significantly prolong PFS without increasing the incidence of grade ≥ 3 adverse events compared with chemo alone. For patients with squamous cell carcinoma, camrelizumab+chemo seems to be a better choice.

## Data availability statement

The raw data supporting the conclusions of this article will be made available by the authors, without undue reservation.

## Author contributions

Conception and design: JL and YiC. Administrative support: TC and YoC. Provision of study materials or patients: HQ and QC. Collection and assembly of data: JQ and YS. Data analysis and interpretation: JL, YiC, and FH. All authors contributed to the article and approved the submitted version.

## Funding

This study was funded by the Western Medicine Guide Project (Grant no. 18411968500) and the Medical Innovation Project (Grant no. 21Y11913500) of the Shanghai Committee of Science and Technology, and the National Key Research and Development Project (No. 2018YFC1705100 and 2018YFC1705103).

## Conflict of interest

The authors declare that the research was conducted in the absence of any commercial or financial relationships that could be construed as a potential conflict of interest.

All authors have completed the ICMJE uniform disclosure form.

## Publisher’s note

All claims expressed in this article are solely those of the authors and do not necessarily represent those of their affiliated organizations, or those of the publisher, the editors and the reviewers. Any product that may be evaluated in this article, or claim that may be made by its manufacturer, is not guaranteed or endorsed by the publisher.

## Abbreviations

NSCLC, non-small cell lung cancer; ICIs, immune checkpoint inhibitors; NMA, network meta-analysis; OS, overall survival; PFS, progression-free survival; AEs, adverse events; PD-L1, programmed cell death ligand-1; PD-1, programmed cell death protein 1; HRs, hazard ratios; ORs, odds ratios; SUCRA, surface under the cumulative ranking; chemo, chemotherapy; Atezo, atezolizumab; Beva, bevacizumab; NK, natural killer.

## References

[1] Sung H Ferlay J Siegel RL Laversanne M Soerjomataram I Jemal A . Global cancer statistics 2020: GLOBOCAN estimates of incidence and mortality worldwide for 36 cancers in 185 countries. CA Cancer J Clin (2021) 71(3):209–249. doi: 10.3322/caac.21660 33538338

[2] Dafni U Tsourti Z Vervita K Peters S . Immune checkpoint inhibitors, alone or in combination with chemotherapy, as first-line treatment for advanced non-small cell lung cancer. a systematic review and network meta-analysis. Lung Cancer (2019) 134:127–40. doi: 10.1016/j.lungcan.2019.05.029 31319971

[3] Sandler A Gray R Perry MC Brahmer J Schiller JH Dowlati A . Paclitaxel–carboplatin alone or with bevacizumab for non–Small-Cell lung cancer. N Engl J Med (2007) 356(3):318. doi: 10.1056/NEJMoa061884 17167137

[4] Zhou C Wu YL Chen G Liu X Zhu Y Lu S . BEYOND: A randomized, double-blind, placebo-controlled, multicenter, phase III study of first-line Carboplatin/Paclitaxel plus bevacizumab or placebo in Chinese patients with advanced or recurrent nonsquamous non-Small-Cell lung cancer. J Clin Oncol (2015) 33(19):2197–204. doi: 10.1200/JCO.2014.59.4424 26014294

[5] Popat S Grohé C Corral J Reck M Novello S Gottfried M . Anti-angiogenic agents in the age of resistance to immune checkpoint inhibitors: Do they have a role in non-oncogene-addicted non-small cell lung cancer? Lung Cancer (2020) 144:76–84. doi: 10.1016/j.lungcan.2020.04.009 32387684

[6] Spigel D de Marinis F Giaccone G Reinmuth N Vergnenegre A Barrios CH . IMpower110: Interim overall survival (OS) analysis of a phase III study of atezolizumab (atezo) vs platinum-based chemotherapy (chemo) as first-line (1L) treatment (tx) in PD-L1–selected NSCLC. Ann Oncol (2019) 30:v915. doi: 10.1093/annonc/mdz293

[7] Reck M Rodriguez-Abreu D Robinson AG Hui R Csőszi T Fulop A . Pembrolizumab versus platinum-based chemotherapy for advanced non–Small-Cell lung cancer with PD-L1 tumor proportion score of 50% or greater. J Clin Oncol (2019) 37(7):537–546. doi: 10.1200/JCO.18 30620668

[8] Gadgeel S Rodr´ıguez-Abreu D Speranza G Esteban E Felip E D´omine M . Updated analysis from KEYNOTE-189: Pembrolizumab or placebo plus pemetrexed and platinum for previously untreated metastatic nonsquamous non–Small-Cell lung cancer. J Clin Oncol (2020) 38(14):1505–17. doi: 10.1200/JCO.19.03136 32150489

[9] Pilotto S Molina-Vila MA Karachaliou N Carbognin L Viteri S González-Cao M . Integrating the molecular background of targeted therapy and immunotherapy in lung cancer: a way to explore the impact of mutational landscape on tumor immunogenicity. Trans Lung Cancer Res (2015) 4(6):721–7. doi: 10.3978/j.issn.2218-6751.2015.10.11 PMC4700230 26798581

[10] Park K Vansteenkiste J Lee KH Pentheroudakis G Zhou C Prabhash H . Pan-Asian adapted ESMO Clinical Practice Guidelines for the management of patients with locally-advanced unresectable non-small-cell lung cancer: a KSMO-ESMO initiative endorsed by CSCO, ISMPO, JSMO, MOS, SSO and TOS. Ann Oncol (2020) 31(2):191–201. doi: 10.1016/j.annonc.2019.10.026 31959336

[11] Hutton B Salanti G Caldwell DM Chaimani A Schmid CH Cameron C . The PRISMA extension statement for reporting of systematic reviews incorporating network meta-analyses of health care interventions: checklist and explanations. Ann Intern Med (2015) 162(11):777–84. doi: 10.7326/M14-2385 26030634

[12] Higgins JP Altman DG Gotzsche PC Juni P Moher D Oxman AD . The cochrane collaboration's tool for assessing risk of bias in randomised trials. BMJ (2011) 343:d5928. doi: 10.1136/bmj.d5928 22008217 PMC3196245

[13] IntHout J Ioannidis JP Borm GF . The hartung-Knapp-Sidik-Jonkman method for random effects meta-analysis is straightforward and considerably outperforms the standard DerSimonian-laird method. BMC Med Res Methodol (2014) 14:25. doi: 10.1186/1471-2288-14-25 24548571 PMC4015721

[14] Nishio M Barlesi F West H Ball S Bordoni R Cobo M . Atezolizumab plus chemotherapy for first-line treatment of nonsquamous NSCLC: Results from the randomized phase 3 IMpower132 trial. J Thorac Oncol (2021) 16(4):653–64. doi: 10.1016/j.jtho.2020.11.025 33333328

[15] Liang J Li M Sui Q Hu Z Bian Y Huang Y . Compare the efficacy and safety of programmed cell death-1 (PD-1) and programmed cell death ligand-1 (PD-L1) inhibitors for advanced non-small cell lung cancer: a Bayesian analysis. Transl Lung Cancer Res (2020) 9(4):1302–23. doi: 10.21037/tlcr-20-192 PMC7481633 32953506

[16] Peters S Ramalingam SS Paz-Ares L Bernabe Caro R Zurawski B Kim S . Nivolumab (NIVO) 1 low-dose ipilimumab (IPI) vs platinumdoublet chemotherapy (chemo) as first-line (1L) treatment (tx) for advanced non-small cell lung cancer (NSCLC): CheckMate 227 part 1 final analysis. Ann Oncol (2019) 30(suppl_5):v851–v934. doi:– 10.1093/annonc/mdz394

[17] Kubota K Yoshioka H Oshita F Hida T Yoh K Hayashi H . Phase III, randomized, placebo-controlled, double-blind trial of motesanib (AMG-706) in combination with paclitaxel and carboplatin in East Asian patients with advanced nonsquamous non–Small-Cell lung cancer. J Clin Oncol (2017) 35(32):3662–3670. doi: 10.1200/JCO.2017.72.7297 28902534

[18] Paz-Ares L Vicente D Tafreshi A Robinson A Soto Parra H Mazières J . Pembrolizumab (pembro) 1 chemotherapy (chemo) in metastatic squamous NSCLC: Final analysis and progression after the next line of therapy (PFS2) in KEYNOTE-407. Ann Oncol (2020) 30(suppl_5):v851–v934. doi: 10.1093/annonc/mdz394

[19] Wang J Lu S Hu C Sun Y Yang K Chen M . Updated analysis of tislelizumab plus chemotherapy vs chemotherapy alone as first-line treatment of advanced squamous non-small cell lung cancer. Annals of Oncology (2020) 31(suppl_4):S754–S840. doi: 10.1016/annonc/annonc283

[20] Socinski MA Jotte RM Cappuzzo F Orlandi F Stroyakovskiy D Nogami N . Atezolizumab for first-line treatment of metastatic nonsquamous NSCLC. N Engl J Med (2018) 378(24):2288–301. doi: 10.1056/NEJMoa1716948 29863955

[21] Zhou C Chen G Huang Y Zhou J Lin L Feng J . Camrelizumab plus carboplatin and pemetrexed versus chemotherapy alone in chemotherapy-naive patients with advanced non-squamous non-small-cell lung cancer (CameL): a randomised, open-label, multicentre, phase 3 trial. Lancet Respir Med (2021) 9(3):305–14. doi: 10.1016/s2213-2600(20)30365-9 33347829

[22] Yang Y Wang Z Fang J Yu Q Han B Cang S . Efficacy and safety of sintilimab plus pemetrexed and platinum as first-line treatment for locally advanced or metastatic nonsquamous NSCLC: a randomized, double-blind, phase 3 study (Oncology pRogram by InnovENT anti-PD-1-11). J Thorac Oncol (2020) 15(10):1636–46. doi: 10.1016/j.jtho.2020.07.014 32781263

[23] West H McCleod M Hussein M Morabito A Rittmeyer A Conter HJ . Atezolizumab in combination with carboplatin plus nab-paclitaxel chemotherapy compared with chemotherapy alone as first-line treatment for metastatic non-squamous non-small-cell lung cancer (IMpower130): A multicentre, randomised, open-label, phase 3 trial. Lancet Oncol (2019) 20(7):924–37. doi: 10.1016/s1470-2045(19)30167-6 31122901

[24] Jotte R Cappuzzo F Vynnychenko I Stroyakovskiy D Rodriguez-Abreu D Hussein M . Atezolizumab in combination with carboplatin and nab-paclitaxel in advanced squamous NSCLC (IMpower131): Results from a randomized phase III trial. J Thorac Oncol (2020) 15(8):1351–60. doi: 10.1016/j.jtho.2020.03.028 32302702

[25] Laurie SA Solomon BJ Seymour L Ellis PM Goss GD Shepherd FA . Randomised, double-blind trial of carboplatin and paclitaxel with daily oral cediranib or placebo in patients with advanced non-small cell lung cancer: NCIC clinical trials group study BR29. Eur J Cancer (2014) 50(4):706–12. doi: 10.1016/j.ejca.2013.11.032 24360368

[26] Scagliotti GV Vynnychenko I Park K Ichinose Y Kubota K Blackhall F . International, randomized, placebo-controlled, double-blind phase III study of motesanib plus carboplatin/paclitaxel in patients with advanced nonsquamous non-small-cell lung cancer: MONET1. J Clin Oncol (2012) 30(23):2829–36. doi: 10.1200/JCO.2011.41.4987 22753922

[27] Ren S Chen J Xu X Jiang T Cheng Y Chen G . CameL-sq Study Group. Camrelizumab Plus Carboplatin and Paclitaxel as First-Line Treatment for Advanced Squamous NSCLC (CameL-Sq): A Phase 3 Trial. J Thorac Oncol (2022) 17(4):544–557. doi: 10.1016/j.jtho.2021.11.018 34923163

[28] Reck M Soconski MA Cappuzzo F Orlandi F Stroyakovskii D Nogami N . Primary PFS and safety analyses of a randomised phase III study of carboplatin + paclitaxel +/– bevacizumab, with or without atezolizumab in 1L non-squamous metastatic NSCLC (IMpower150). Annals of Oncology (2017) 28(suppl_11. doi: 10.1093/annonc/mdx760.002

[29] Wang J Lu S Hu C Sun Y Yang K Chen M . 1264P updated analysis of tislelizumab plus chemotherapy vs chemotherapy alone as first-line treatment of advanced squamous non-small cell lung cancer (SQ NSCLC). Ann Oncol (2020) 31(suppl_4):S754–S840. doi: 10.1016/j.annonc.2020.08.1578

[30] Paz-Ares L Vicente D Tafreshi A Robinson A Soto Parra H Mazieres J . A randomized, placebo-controlled trial of pembrolizumab plus chemotherapy in patients with metastatic squamous NSCLC: Protocol-specified final analysis of KEYNOTE-407. J Thorac Oncol (2020) 15(10):1657–69. doi: 10.1016/j.jtho.2020.06.015 32599071

[31] Sugawara S Lee JS Kang JH Kim HR Inui N Hida T . Nivolumab with carboplatin, paclitaxel, and bevacizumab for first-line treatment of advanced nonsquamous non-small-cell lung cancer. Ann Oncol (2021) 32(9):1137–47. doi: 10.1016/j.annonc.2021.06.004 34139272

[32] Tran E Turcotte S Gros A Robbins PF Lu YC Dudley ME . Cancer immunotherapy based on mutation-specific CD4+ T cells in a patient with epithelial cancer. Science (2014) 344(6184):641–5. doi: 10.1126/science.1251102 PMC6686185 24812403

[33] Boussiotis VA . Somatic mutations and immunotherapy outcome with CTLA-4 blockade in melanoma. N Engl J Med (2014) 371(23):2230–2. doi: 10.1056/NEJMe1413061 PMC4456677 25409261

[34] Latchman YE Liang SC Wu Y Chernova T Sobel RA Klemm M . PD-L1-deficient mice show that PD-L1 on T cells, antigen-presenting cells, and host tissues negatively regulates T cells. Proc Natl Acad Sci U S A (2004) 101(29):10691–6. doi: 10.1073/pnas.0307252101 PMC489996 15249675

[35] Dong W Wu X Ma S Wang Y Nalin AP Zhu Z . The mechanism of anti-PD-L1 antibody efficacy against PD-L1-Negative tumors identifies NK cells expressing PD-L1 as a cytolytic effector. Cancer Discovery (2019) 9(10):1422–37. doi: 10.1158/2159-8290.CD-18-1259 PMC7253691 31340937

[36] Caligiuri M . Natural killer cells in innate immunity and cancer. J Immunother (2008) 31(8):685–92. doi: 10.1097/CJI.0b013e318182de23 18779751

[37] Cong J Wei H . Natural killer cells in the lungs. Front Immunol (2019) 10:1416. doi: 10.3389/fimmu.2019.01416 31293580 PMC6603080

[38] Duan J Cui L Zhao X Bai H Cai S Wang G . Use of immunotherapy with programmed cell death 1 vs programmed cell death ligand 1 inhibitors in patients with cancer a systematic review and meta-analysis. JAMA Oncol(2020) 6(3):375–384. doi: 10.1001/jamaoncol.2019.5367 PMC6990765 31876895

[39] Onesti CE Freres P Jerusalem G . Atypical patterns of response to immune checkpoint inhibitors: interpreting pseudoprogression and hyperprogression in decision making for patients' treatment. J Thorac Dis (2019) 11(1):35–8. doi: 10.21037/jtd.2018.12.47 PMC6384391 30863564

[40] Borghaei H Paz-Ares L Horn L Spigel DR Steins M Ready NE . Nivolumab versus docetaxel in advanced nonsquamous non-Small-Cell lung cancer. N Engl J Med (2015) 373(17):1627–39. doi: 10.1056/NEJMoa1507643 PMC5705936 26412456

