# Supplementary material for: Comparison of the efficacy and safety in the treatment strategies between chemotherapy combined with antiangiogenic and with immune checkpoint inhibitors in advanced non-small cell lung cancer patients with negative PD-L1 expression: A network meta-analysis
Source: Front Oncol. 2022 Nov 29;12:1001503. doi: 10.3389/fonc.2022.1001503 (PMC9746688; doi:10.3389/fonc.2022.1001503)
Supplement: Supplementary file 1 [file DataSheet_1.pdf]

# Comparison of the efficacy and safety in the treatment strategies between chemotherapy combined with antiangiogenic and with immune checkpoint inhibitors in advanced non-small cell lung cancer patients with negative PD-L1 expression: A network meta-analysis

## Supplementary materials

((randomized controlled trial[Publication Type] OR randomized[Title/Abstract] OR placebo[Title/Abstract]) AND (((((((((((((((((((((((Immunotherapy[MeSH Terms]) OR (PD-1[Title/Abstract])) OR (PD-L1[Title/Abstract])) OR (CTLA-4[Title/Abstract])) OR (Pembrolizumab[Title/Abstract])) OR (Keytruda[Title/Abstract])) OR (lambrolizumab[Title/Abstract])) OR (MK-3475[Title/Abstract])) OR (Nivolumab[Title/Abstract])) OR (Atezolizumab[Title/Abstract])) OR (Tecentriq[Title/Abstract])) OR (MPDL3280A[Title/Abstract])) OR (Avelumab[Title/Abstract])) OR (Bavencio[Title/Abstract])) OR (Durvalumab[Title/Abstract])) OR (Imfinzi[Title/Abstract])) OR (Camrelizumab[Title/Abstract])) OR (Tislelizumab[Title/Abstract])) OR (Sintilimab[Title/Abstract])) OR (ipilimumab[Title/Abstract])) OR (tremelimumab[Title/Abstract])) OR (ticilimumab[Title/Abstract])) OR ((((((((((((((((((((((((((((Angiogenesis Inhibitors[MeSH Terms]) OR (Inhibitor*,Angiogen*[Title/Abstract])) OR (Antagonist* Angiogen*[Title/Abstract])) OR (Angiostatic Agent*[Title/Abstract])) OR (Anti？ Angiogen* Agent*[Title/Abstract])) OR (Anti？ Angiogen* Drug*[Title/Abstract])) OR (Inhibitor*, Neovascularization[Title/Abstract])) OR (VEGFR[Title/Abstract])) OR (multitargeted antiangiogenesis tyrosine kinase inhibitors[Title/Abstract])) OR (*mab[Title/Abstract])) OR (*nib[Title/Abstract])) OR (BIBF 1120[Title/Abstract])) OR (votrient[Title/Abstract])) OR (Everolimus[Title/Abstract])) OR (Aflibercept[Title/Abstract])) OR (bevacizumab[Title/Abstract])) OR (ramucirumab[Title/Abstract])) OR (nintedanib[Title/Abstract])) OR (anlotinib[Title/Abstract])) OR (apatinib[Title/Abstract])) OR (Sorafenib[Title/Abstract])) OR (Vandetanib[Title/Abstract])) OR (Sunitinib[Title/Abstract])) OR (Pazopanib[Title/Abstract])) OR (Lenvatinib[Title/Abstract])) OR (Cediranib[Title/Abstract])) OR (Motesanib[Title/Abstract])) OR (Axitinib[Title/Abstract])))) AND ((((Carcinoma, Non-Small-Cell Lung[MeSH Terms]) OR (Lung Carcinoma*, Non-Small-Cell[[Title/Abstract])) OR (Non?Small ?Cell Lung Cancer[Title/Abstract])) OR (NSCLC[Title/Abstract]))

**Table S1. Literature search strategy on pubmed.**

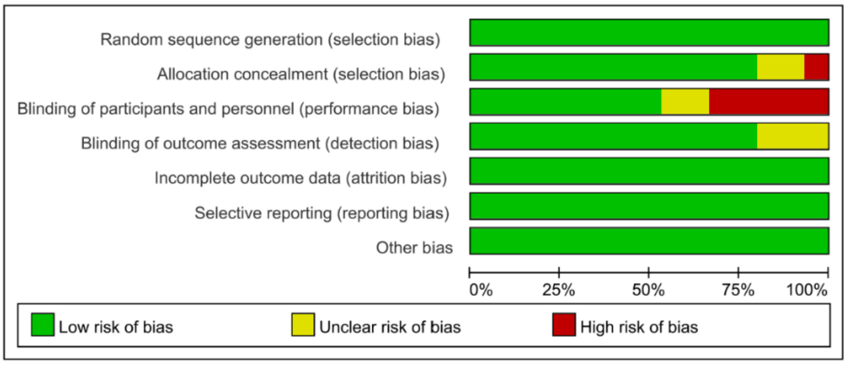


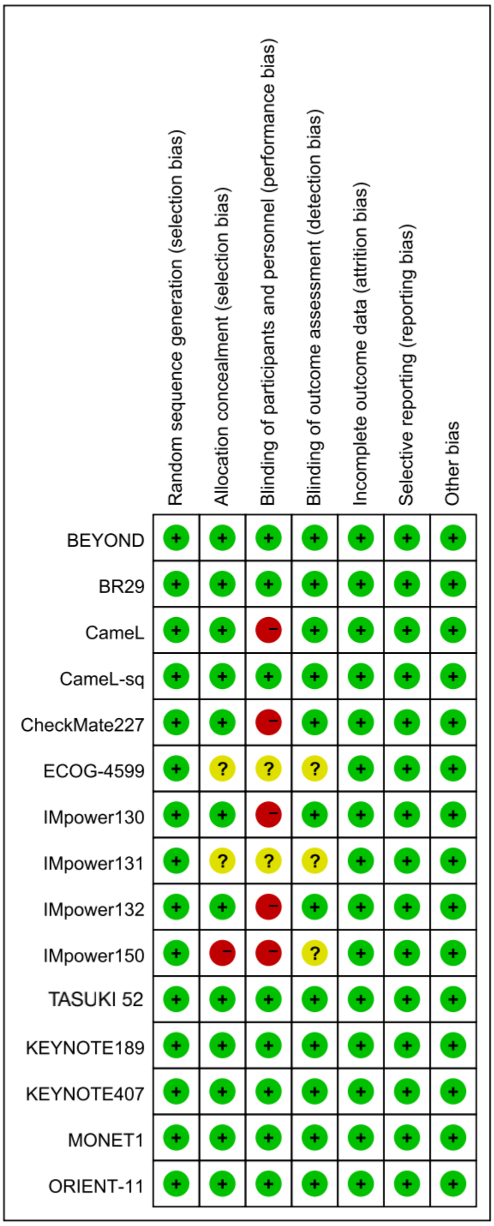


Figure S1 Assessment of risk of bias

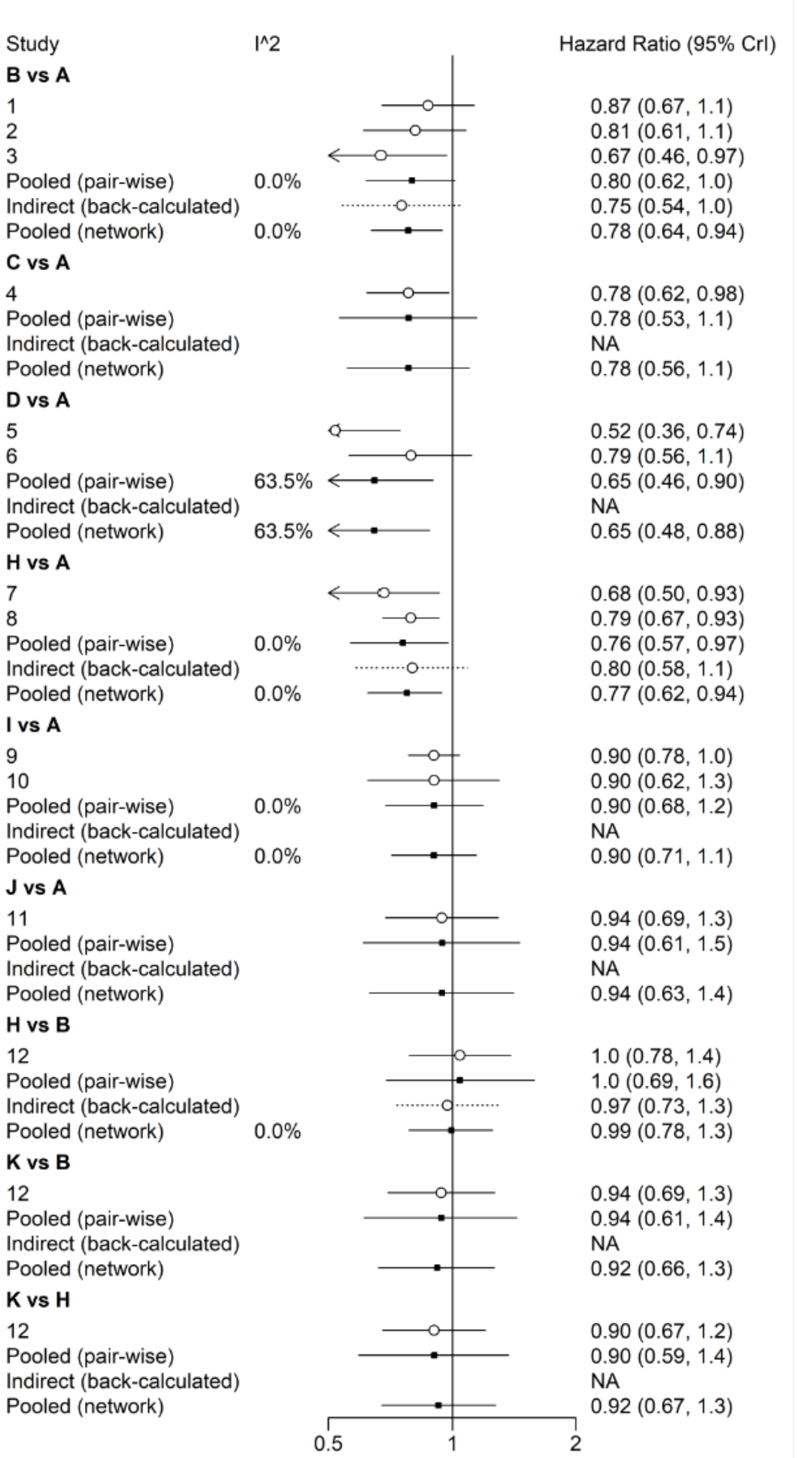


A: Forest plots of pairwise comparisons with heterogeneity estimates for OS

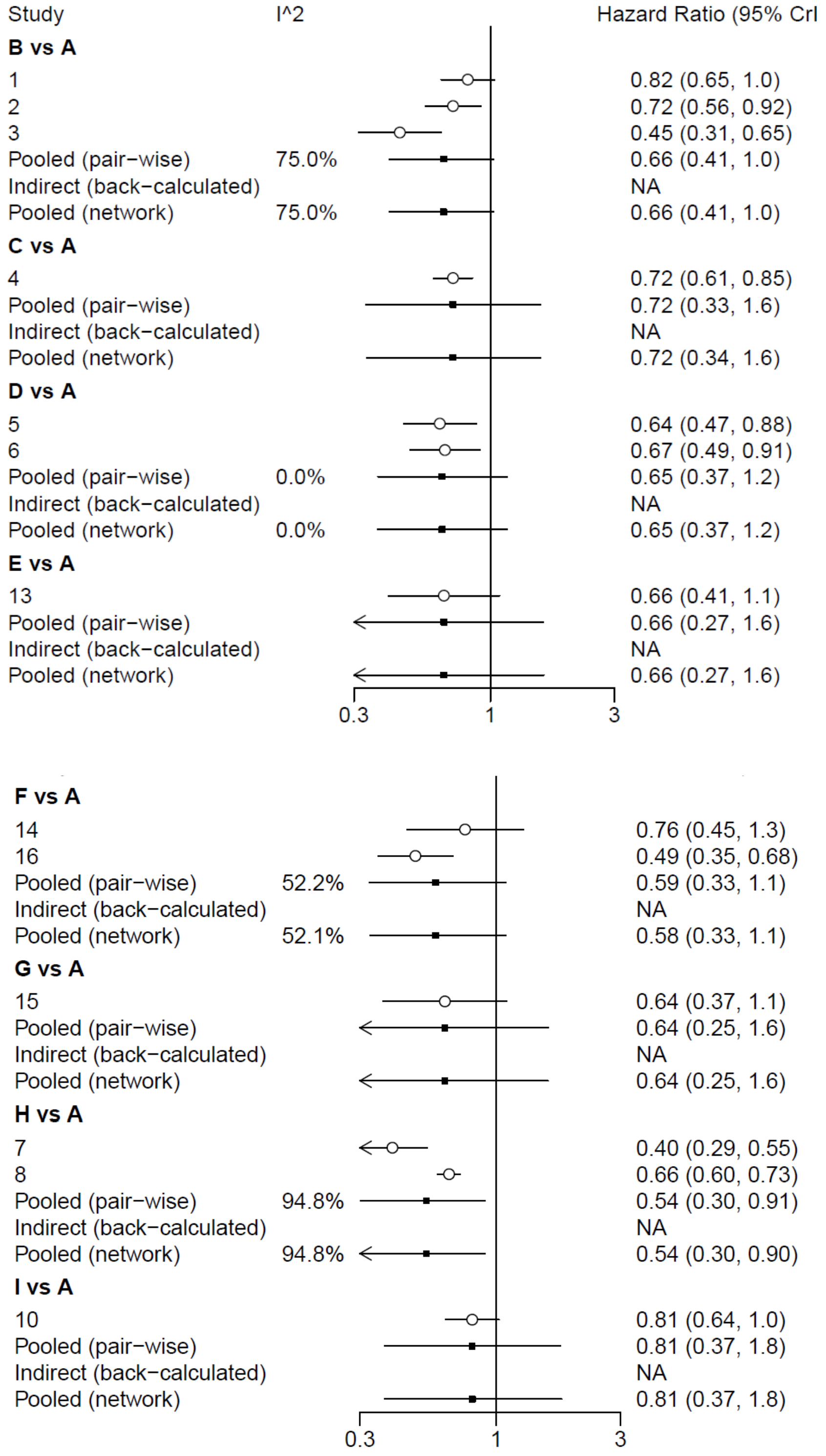
Study
I^2
Hazard Ratio (95% CrI
B vs A
1 0.82 (0.65, 1.0)
2 0.72 (0.56, 0.92)
3 0.45 (0.31, 0.65)
Pooled (pair−wise) 75.0% 0.66 (0.41, 1.0)
Indirect (back−calculated) NA
Pooled (network) 75.0% 0.66 (0.41, 1.0)
C vs A
4 0.72 (0.61, 0.85)
Pooled (pair−wise) 0.72 (0.33, 1.6)
Indirect (back−calculated) NA
Pooled (network) 0.72 (0.34, 1.6)
D vs A
5 0.64 (0.47, 0.88)
6 0.67 (0.49, 0.91)
Pooled (pair−wise) 0.0% 0.65 (0.37, 1.2)
Indirect (back−calculated) NA
Pooled (network) 0.0% 0.65 (0.37, 1.2)
E vs A
13 0.66 (0.41, 1.1)
Pooled (pair−wise) 0.66 (0.27, 1.6)
Indirect (back−calculated) NA
Pooled (network) 0.66 (0.27, 1.6)
0.3
1
3
F vs A
14 0.76 (0.45, 1.3)
16 0.49 (0.35, 0.68)
Pooled (pair−wise) 52.2% 0.59 (0.33, 1.1)
Indirect (back−calculated) NA
Pooled (network) 52.1% 0.58 (0.33, 1.1)
G vs A
15 0.64 (0.37, 1.1)
Pooled (pair−wise) 0.64 (0.25, 1.6)
Indirect (back−calculated) NA
Pooled (network) 0.64 (0.25, 1.6)
H vs A
7 0.40 (0.29, 0.55)
8 0.66 (0.60, 0.73)
Pooled (pair−wise) 94.8% 0.54 (0.30, 0.91)
Indirect (back−calculated) NA
Pooled (network) 94.8% 0.54 (0.30, 0.90)
I vs A
10 0.81 (0.64, 1.0)
Pooled (pair−wise) 0.81 (0.37, 1.8)
Indirect (back−calculated) NA
Pooled (network) 0.81 (0.37, 1.8)
0.3
1
3

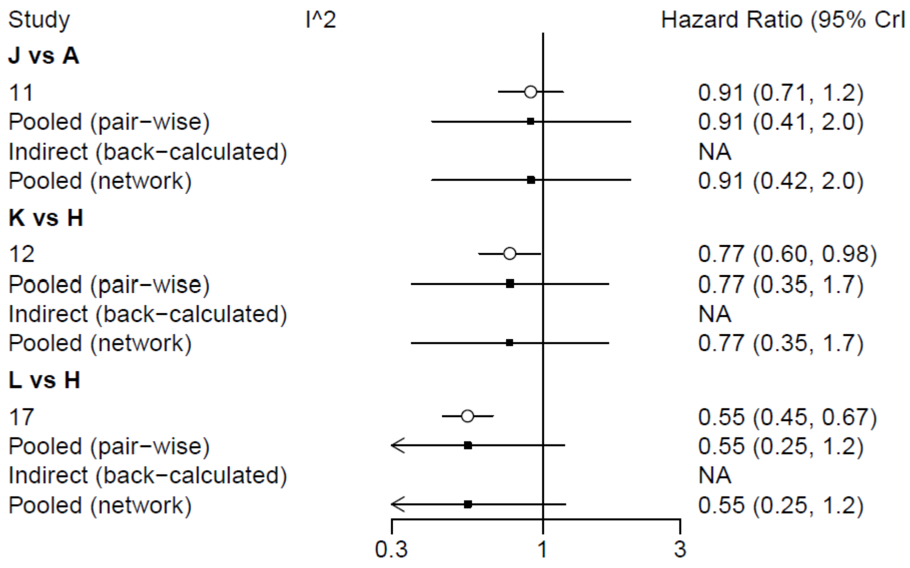


B: Forest plots of pairwise comparisons with heterogeneity estimates for PFS.

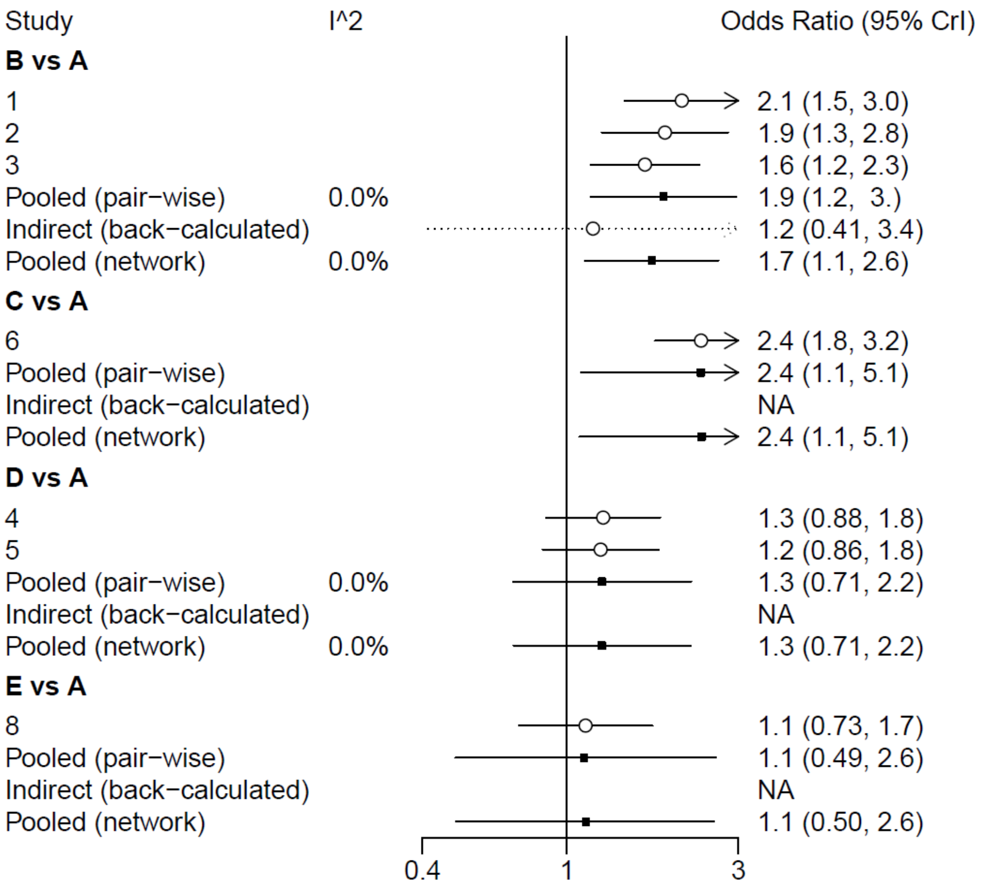
Study
I^2
Odds Ratio (95% CrI)
B vs A
1 2.1 (1.5, 3.0)
2 1.9 (1.3, 2.8)
3 1.6 (1.2, 2.3)
Pooled (pair−wise) 0.0% 1.9 (1.2, 3.)
Indirect (back−calculated) 1.2 (0.41, 3.4)
Pooled (network) 0.0% 1.7 (1.1, 2.6)
C vs A
6 2.4 (1.8, 3.2)
Pooled (pair−wise) 2.4 (1.1, 5.1)
Indirect (back−calculated) NA
Pooled (network) 2.4 (1.1, 5.1)
D vs A
4 1.3 (0.88, 1.8)
5 1.2 (0.86, 1.8)
Pooled (pair−wise) 0.0% 1.3 (0.71, 2.2)
Indirect (back−calculated) NA
Pooled (network) 0.0% 1.3 (0.71, 2.2)
E vs A
8 1.1 (0.73, 1.7)
Pooled (pair−wise) 1.1 (0.49, 2.6)
Indirect (back−calculated) NA
Pooled (network) 1.1 (0.50, 2.6)
0.4
1
3

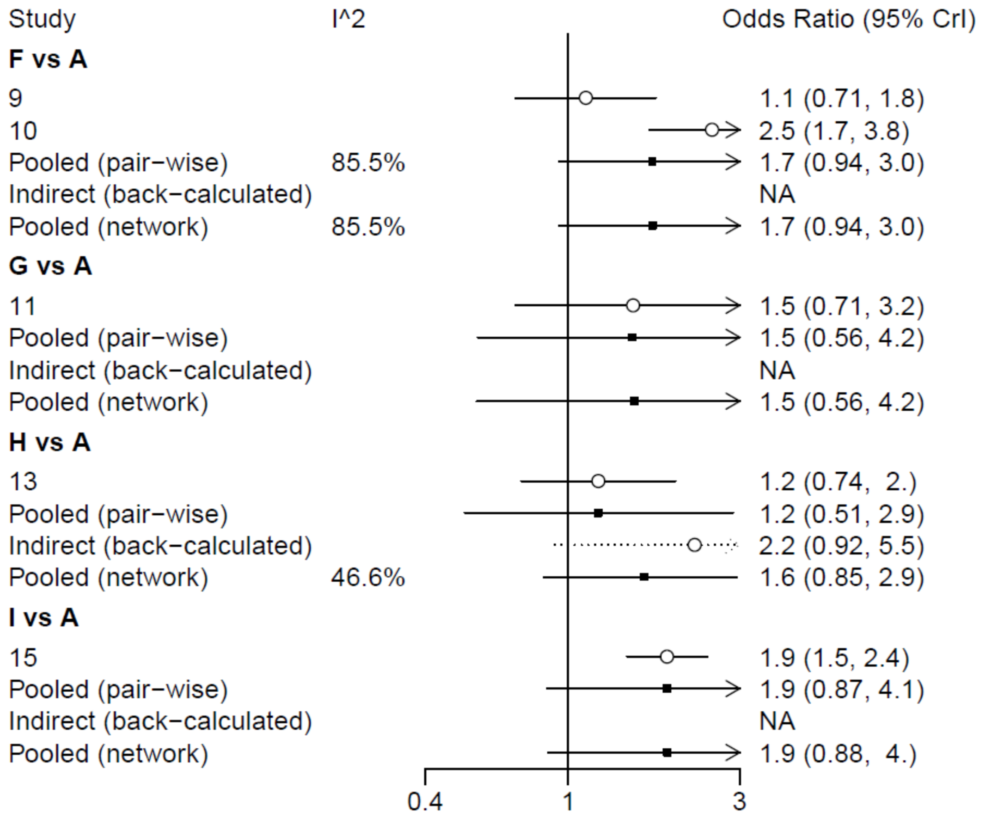
Study
I^2
Odds Ratio (95% CrI)
F vs A
9 1.1 (0.71, 1.8)
10 2.5 (1.7, 3.8)
Pooled (pair−wise) 85.5% 1.7 (0.94, 3.0)
Indirect (back−calculated) NA
Pooled (network) 85.5% 1.7 (0.94, 3.0)
G vs A
11 1.5 (0.71, 3.2)
Pooled (pair−wise) 1.5 (0.56, 4.2)
Indirect (back−calculated) NA
Pooled (network) 1.5 (0.56, 4.2)
H vs A
13 1.2 (0.74, 2.)
Pooled (pair−wise) 1.2 (0.51, 2.9)
Indirect (back−calculated) 2.2 (0.92, 5.5)
Pooled (network) 46.6% 1.6 (0.85, 2.9)
I vs A
15 1.9 (1.5, 2.4)
Pooled (pair−wise) 1.9 (0.87, 4.1)
Indirect (back−calculated) NA
Pooled (network) 1.9 (0.88, 4.)
0.4
1
3

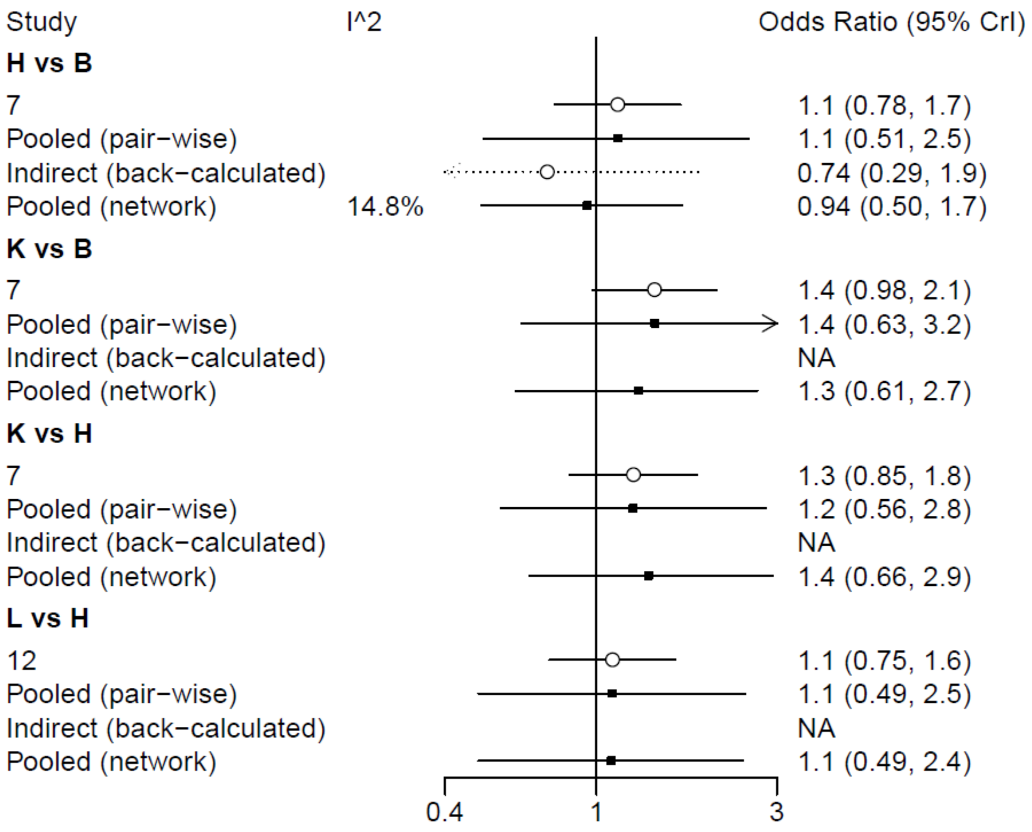


C: Forest plots of pairwise comparisons with heterogeneity estimates for safety(⩾3AE)

**Figure S2: Forest plots of pairwise comparisons with heterogeneity.**

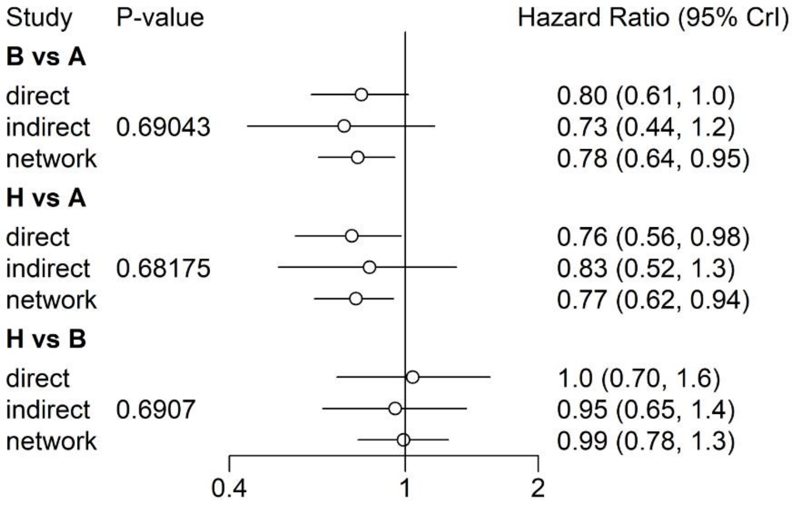


A: Node-splitting analysis of inconsistency for OS

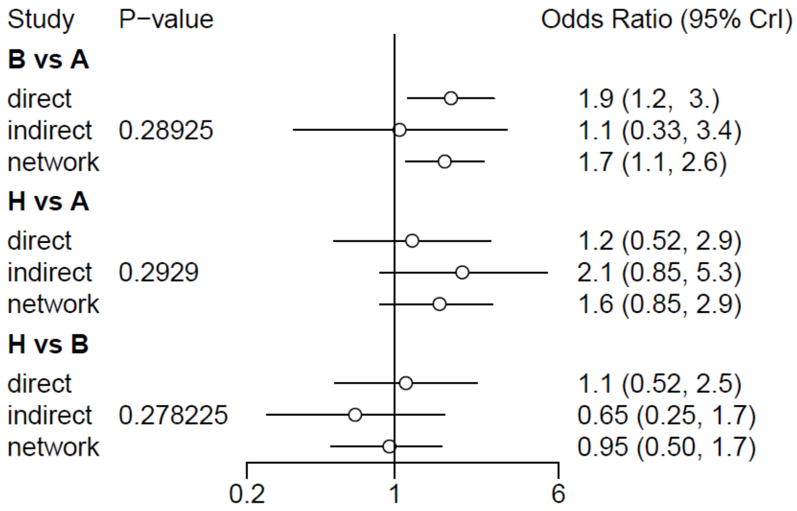


B: Node-splitting analysis of inconsistency for PFS

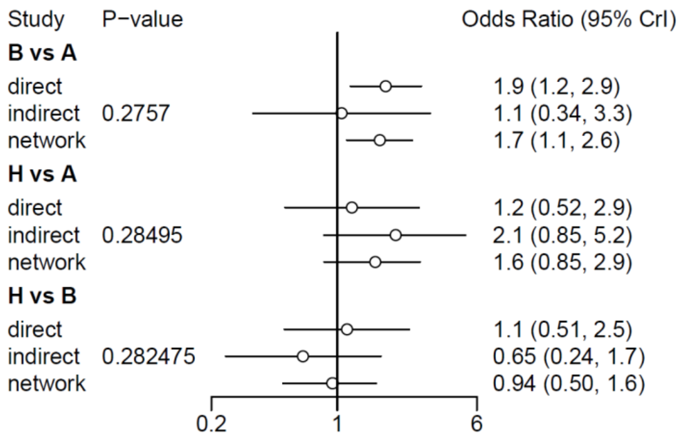


C: Node-splitting analysis of inconsistency for safety(≥3AE)

**Figure S3: Node-splitting analysis of inconsistency.**

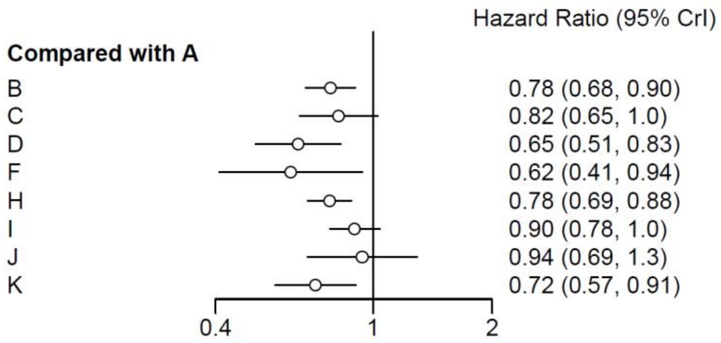

A:Forest plot for OS

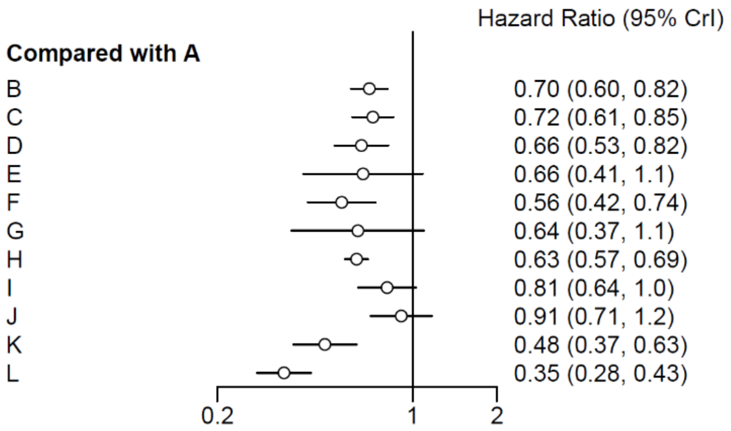


B: Forest plot for PFS

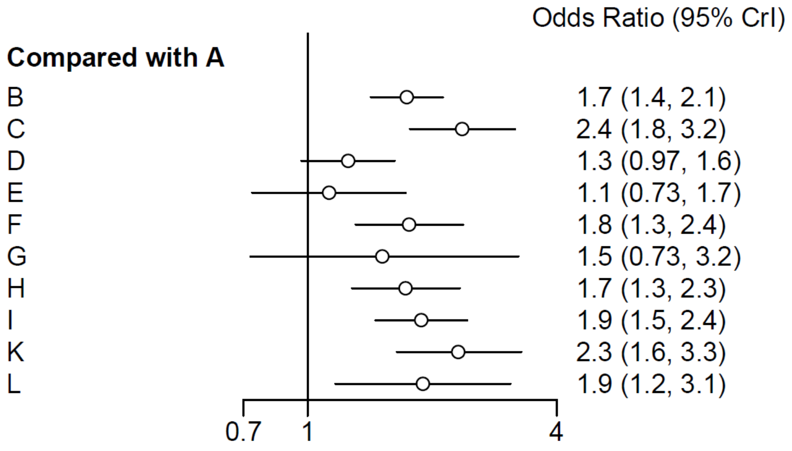


C: Forest plot for ≥3AE

**Figure S4: Forest plots**

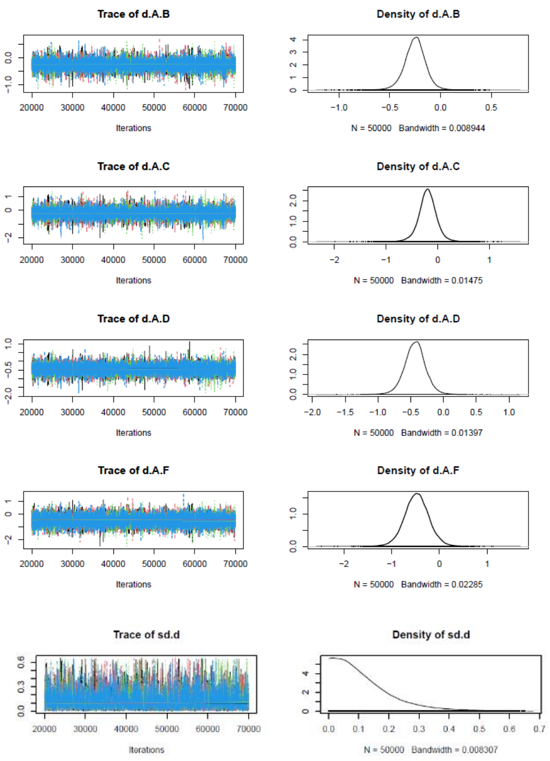


A:Trace and density plots for OS

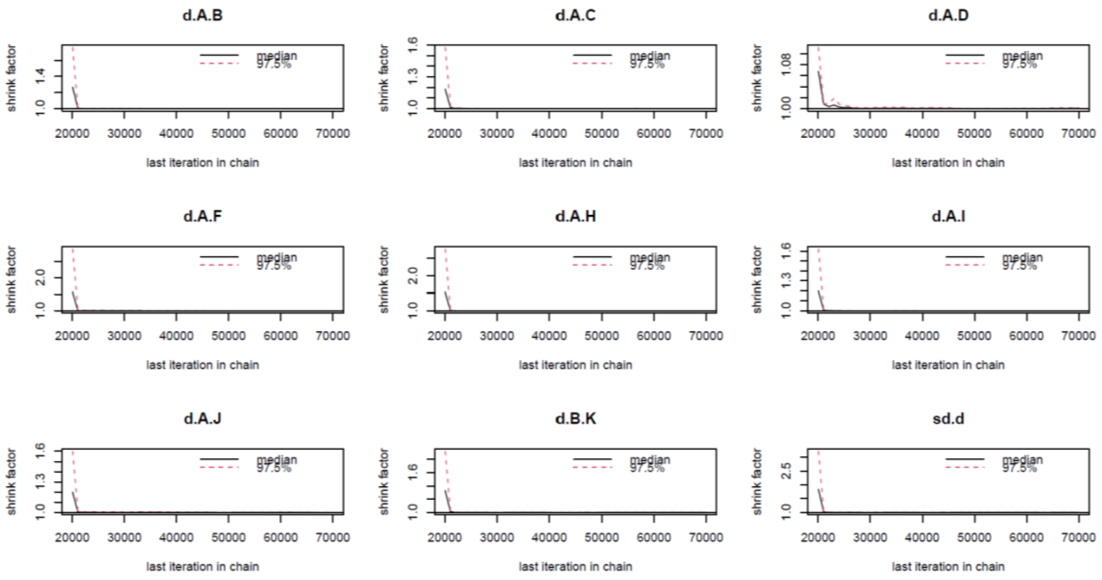


B: Brooks-Gelman-Rubin diagnosis plot for OS

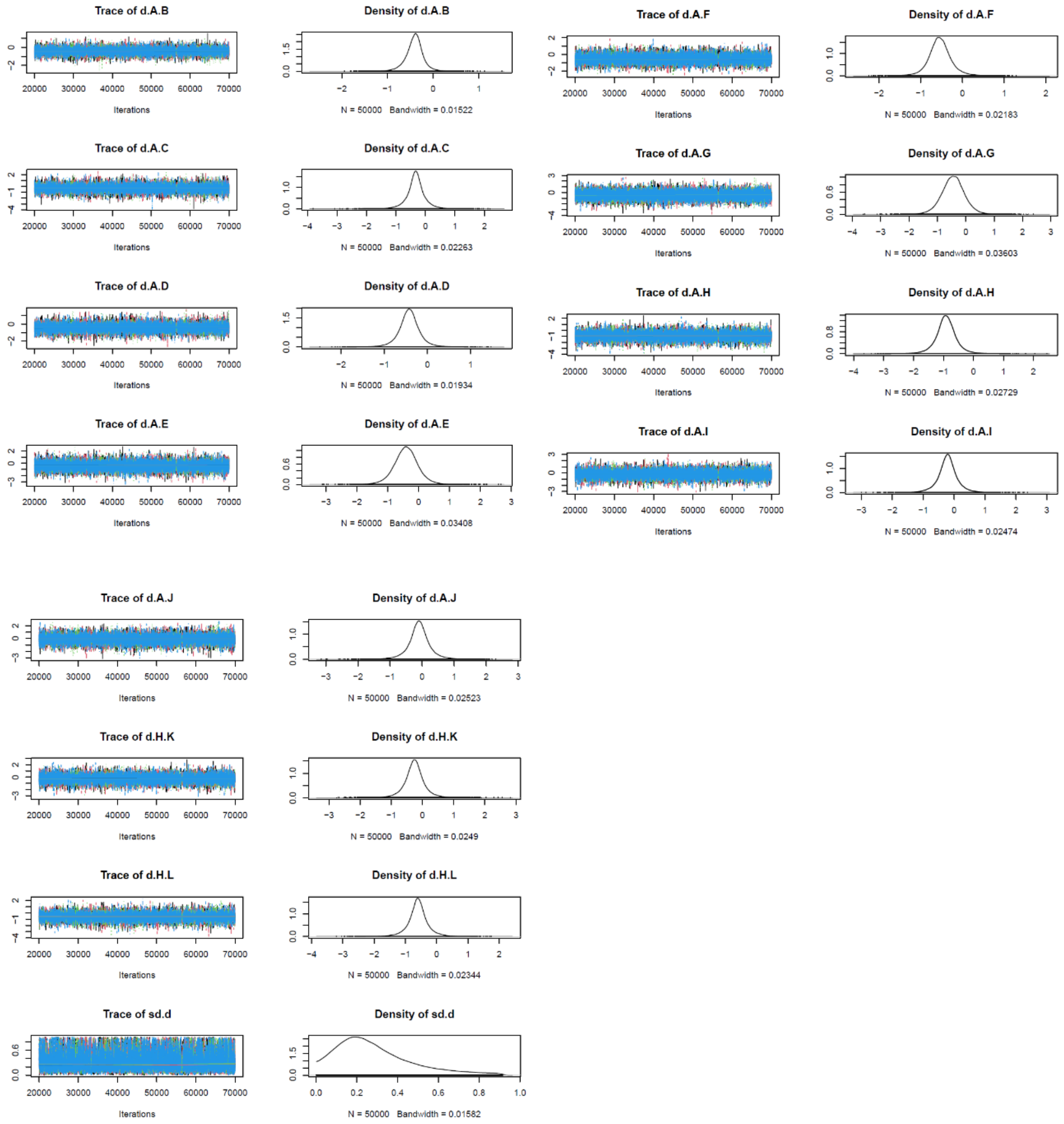


C:Trace and density plots for PFS

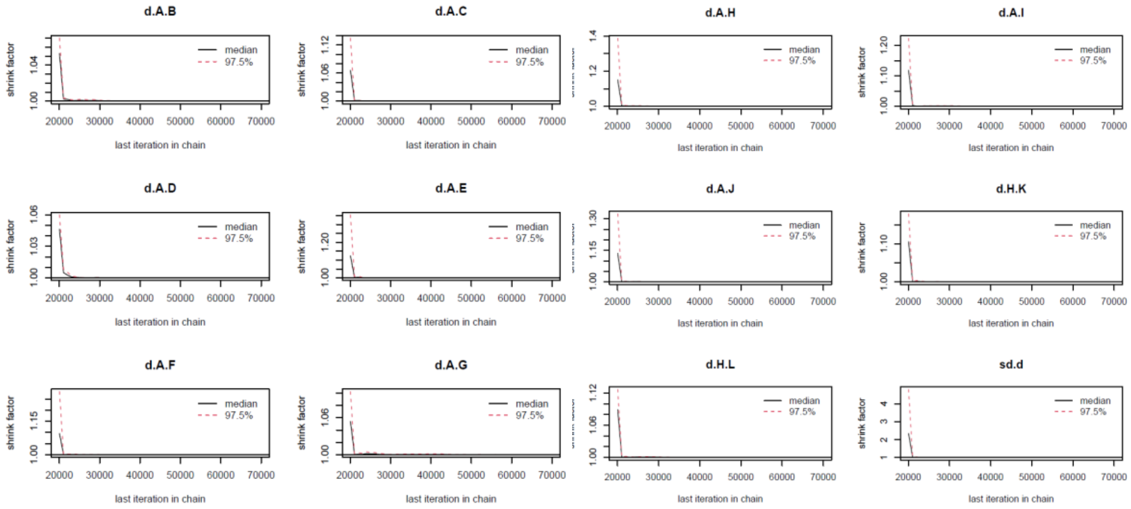


D: Brooks-Gelman-Rubin diagnosis plot for PFS

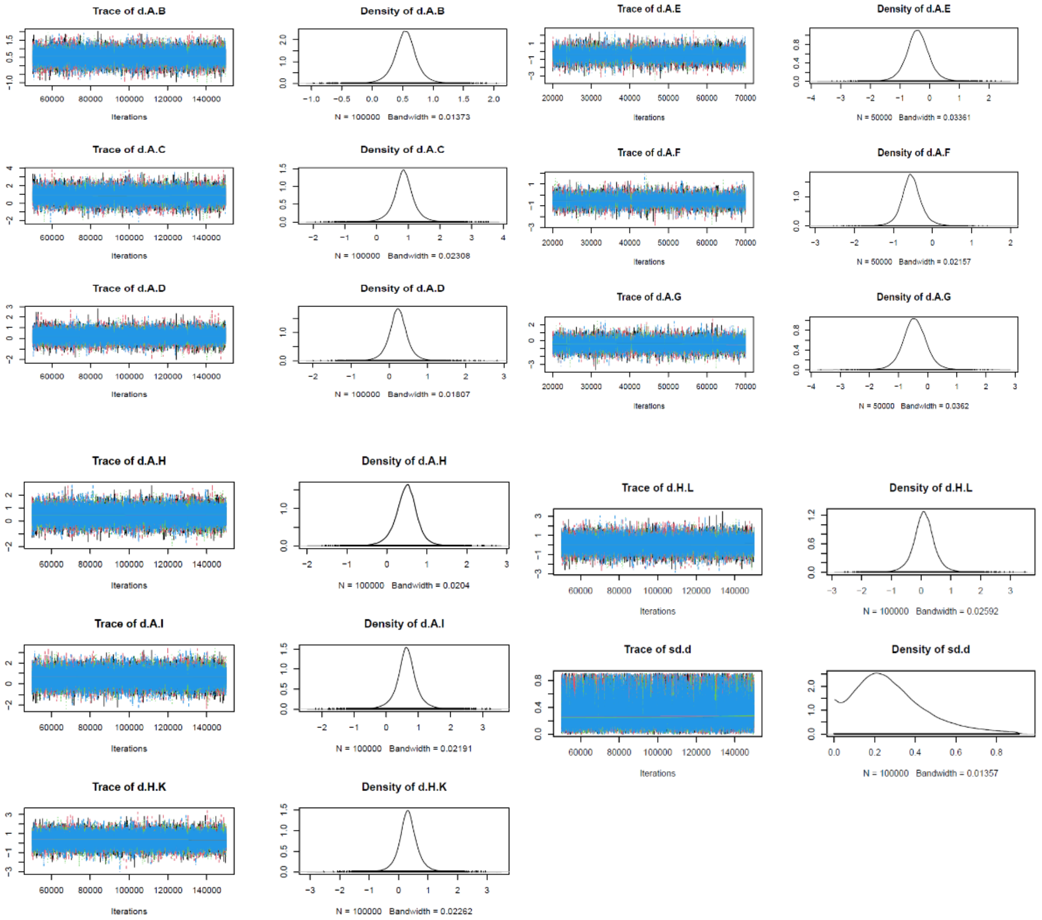


E:Trace and density plots for grade ⩾3 adverse events

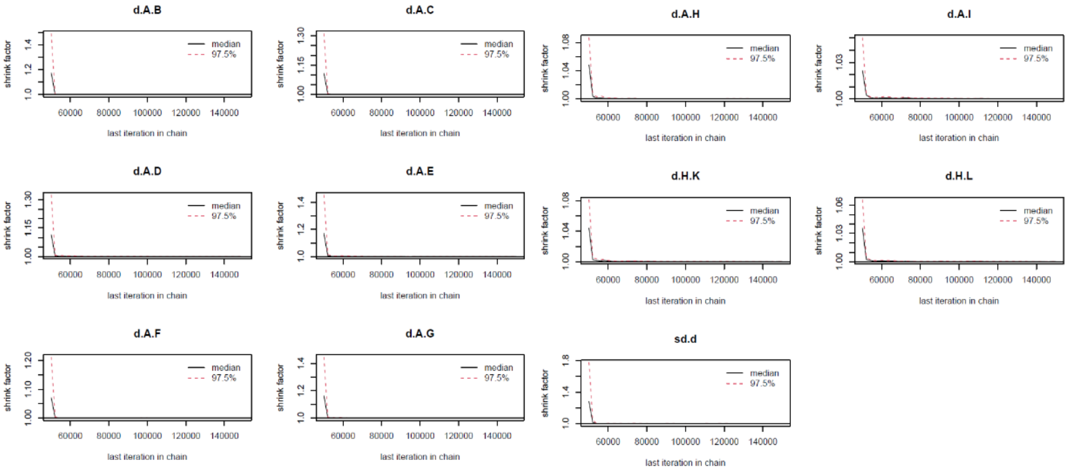


F: Brooks-Gelman-Rubin diagnosis plot for grade ≥3 adverse events

**Figure S5: Trace and density plots and the Brooks Gelman-Rubin diagnostic for OS (A and B), PFS (C and D) and grade ≥3 adverse events (E and F).**
